# Crowding for Confinement: Reversible Isomerization of First-Generation Donor-Acceptor Stenhouse Adduct Derivatives in Water Modulated by Thermoresponsive Dendritic Macromolecules

**DOI:** 10.3390/molecules29215055

**Published:** 2024-10-26

**Authors:** Jiaxing Zhang, Qinqin Ma, Huan Wang, Peinan Zhang, Xinyan Su, Afang Zhang, Wen Li

**Affiliations:** International Joint Laboratory of Biomimetic and Smart Polymers, School of Materials Science and Engineering, Shanghai University, Mailbox 152, Shangda Rd. 99, Shanghai 200444, China

**Keywords:** dendrimers, dendronized polymers, thermoresponsive polymers, donor-acceptor Stenhouse adducts, isomerization, crowding, confinement

## Abstract

Mimicking nature, the reversible isomerization of hydrophobic dyes in aqueous solutions is appealing for bio-applications. Here, we report on the reversible isomerization of first-generation solvatochromic donor-acceptor Stenhouse adducts (DASAs) in water within dendritic matrices, realized either through the dendronization of DASAs or the incorporation of DASA pendants into dendronized copolymers. These dendritic macromolecules contain three-fold dendritic oligoethylene glycols (OEGs), which afford the macromolecules water-solubility and unprecedented thermoresponsive behavior. The thermoresponsive behavior of both dendronized DASAs and dendronized copolymers is dominated by the peripherals of dendritic OEGs. However, the hydrophilicity of the acceptor from DASA moieties also play a role in mediating their thermal phase transitions, and more importantly, tailor the hydrophobic interactions between dendritic OEGs and DASA moieties. Intriguingly, dendritic topologies contribute confinement to encapsulate the DASA moieties through crowding effects, and cooperative interactions from the crowded dendritic OEGs modulate the DASA moieties with different isomerization in aqueous media. The thermally induced collapse of dendritic OEGs, accompanied by the aggregation of dendritic macromolecules, leads to the formation of hydrophobic domains, which exert enhanced crowding effects to efficiently encapsulate the DASA moieties. Compared to the low molar mass of dendronized DASAs, thermally collapsed dendronized copolymers can efficiently retard the hydration of DASA pendants through cooperation between neighboring dendritic OEGs and afford the DASA pendants with better confined microenvironments to mediate their isomerization recovery by up to 90% from a cyclic charged (hydrophilic) state into a noncharged (hydrophobic) linear state in water. This dendritic confinement exhibits excellent fatigue resistance after several cycles of alternating photo-irradiation and thermal annealing at elevated temperatures.

## 1. Introduction

Reversible photoisomerization in nature plays an important role in energy transformation and color changes, which is always realized in aqueous environments [1,2,3]. For example, a sunflower disk shifts its direction with the angle of the sun [4], chameleons change their skin color depending on environmental temperature and light intensity [5,6], and green plants absorb sunlight and convert light energy into chemical energy to support the world [7,8,9]. By mimicking nature, the photoisomerization of synthetic (macro)molecules is appealing, not only for principal exploration, but also for the development of optical-responsive intelligent materials. Up to dates, a series of photosensitive molecules, such as azobenzene, spiropyran, and diarylethene, have been extensively investigated [10,11,12,13] and have found promising applications in various fields [14,15,16]. However, conventional photosensitive molecules are usually lipophilic and can only be dissolved in organic solvents to regulate their isomerization, which limits their practical applications in biomedicines and biomaterials [17,18,19]. In addition, the successful operation of an optical switch in aqueous media requires both hydrolytic stability and biological applicability in photosensitive dyes to a certain degree, while maintaining photochromic and reversible isomerization characteristics. To date, to make lipophilic dyes accessible in aqueous media, different strategies have been applied to improve their water solubility, including molecular cage encapsulation [20,21], molecular structural modifications [22,23], and supramolecular self-assembly [24,25]. However, the reversible isomerization of these photosensors in a polar water environment still remains the toughest challenge due to the difficulty in tailoring the energy balance between their different isomers. In addition, these methodologies are applicable only to selective dyes, partially due to either strictly spatial requirements or insufficient interaction strengths. In fact, the microconfinement of biomacromolecules resulting from molecular crowding imposes constraints on their well-defined biological functions and activities. To replicate this phenomenon, synthetic polymers that form restricted structures or microenvironments are of significant scientific importance and have recently garnered considerable attention. The successful creation of constrained microenvironments from synthetic macromolecules relies on the application of molecular crowding effects and topological synergies, with a key emphasis on striking a balance between the self-incorporation of structural units and the self-exclusion of spatial-site resistance induced by crowding [26,27,28,29].

Donor-acceptor Stenhouse adducts (DASAs) offer the benefits of extended wavelength absorption, robust fatigue resistance, and efficient photo-stationary state conversion between two isomers, simultaneously accompanied by color changes among visible wavelengths [30,31,32]. When alternately exposed to visible light and heating, DASAs can effectively undergo reversible transformations between the linear (lipophilic) and cyclic (hydrophilic) states [33,34,35]. First-generation DASAs utilize an alkyl-chain secondary amine as the donor motif and 1,3-dimethylbarbituric acid as the acceptor motif [36]. Their linear open-loop structure exhibits a higher dipole moment compared to a zwitterionic ring structure. In dichloromethane (DCM), most of them exist stably as trienes (red-colored) in the thermal equilibrium state; however, their spectral absorption range is narrow, and reversible transformation can only be achieved in toluene and other nonpolar aromatic solvents [37,38,39]. Differently, third-generation DASAs incorporate new receptor elements, primarily pyrazolidinone, isoxazolidone, and indandione, and thereby increase compound diversity and broaden spectral absorption range while demonstrating far-infrared photoactivity through isomerization between two non-charged isomers [40,41,42,43]. Furthermore, these modifications enhance the stability of the open-loop structure and control the thermal equilibrium rate by shortening closed-open-loop isomerization time [44,45,46]. However, due to a highly polarized zwitterionic ring structure, it remains a great challenge to control the reversible isomerization of the first generation of DASAs in a polar solvent like water, mainly because the zwitterionic isomer endows extensive hydration in water, making it difficult to recover back into a hydrophobic, non-ionic linear state [47,48,49].

**Figure 1:**
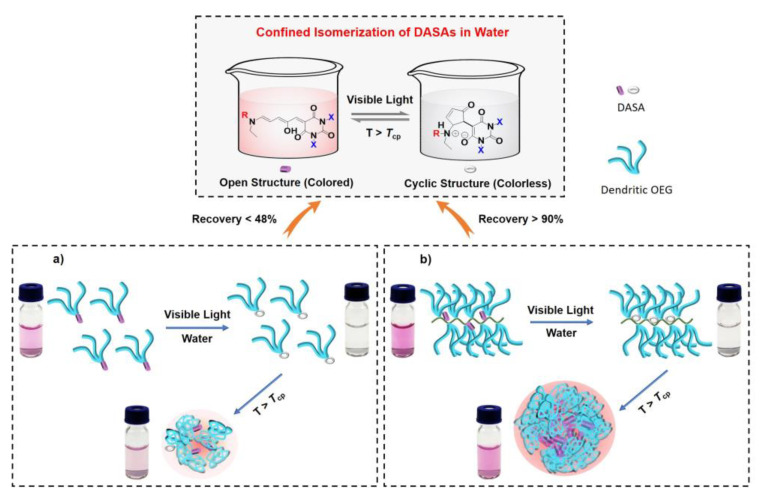
Illustration of confinement from dendritic OEGs to mediate reversible isomerization of DASAs in water. DASA moieties were connected to three-fold dendritic OEGs (**a**) or dendronized copolymethacrylates carrying three-fold dendritic OEGs (**b**), which exhibited characteristic thermoresponsive behavior. Thermally induced dehydration and collapse of dendritic OEGs formed hydrophobic domains, which mediated isomerization recovery for DASAs in water from hydrophilic cyclic states into conjugated linear states with varying efficiency.

A homologous series of dendritic macromolecules were developed by us previously. They carried dendritic oligoethylene glycol (OEG) pendants of different branching densities and had different polymeric backbones, including dendronized polymethacrylates [50], dendronized chitosans [51], and helical dendronized poly(phenylene)s [52]. These polymers exhibited unprecedented thermoresponsive behavior, with fast and sharp phase transitions. In addition, due to the thickness of these worm-like macromolecules, they underwent heterogeneous thermal dehydration, affording “molecular envelop” effects with peripherals dehydrated at low temperatures, and the interiors dehydrated at much higher temperatures [53]. This feature provides an intriguing opportunity to tailor the molecular crowding effect with the selective encapsulation of dyes, whose physical states can be mediated [54]. In addition, this “molecular envelop” effect has been successfully applied to encapsulate biomacromolecules such as proteins or RNA, modulate their bioactivities, and protect them from degradation by enzymes. Interestingly, low-molar-mass dendritic macromolecules carrying dendritic OEGs were found to also exhibit typical thermoresponsive behavior. Encouraged by these findings, we recently reported the dendronization of third generation DASAs with three- or six-fold dendritic OEGs and investigated their reversible isomerization in water through dendritic confinement, finding that the isomerization of these DASA moieties within the matrices of dendritic OEGs can be modulated reversibly to a great extent [55]. Based on these achievements, we target in this study the challenge of modulating the reversible isomerization of first-generation DASAs in water through dendritic confinement. Two different levels of dendritic confinements were designed for this purpose: through dendronization of the DASAs with dendritic OEGs to form low-molar-mass spherical dendronized DASAs, and through the incorporation of DASA moieties into high-molar-mass, worm-like dendronized copolymers that densely carried the dendritic OEG pendants. The dendronization of DASAs with dendritic OEGs afforded these dyes water-solubility and allowed for the investigation of their photo-isomerization in aqueous media. The structural effects on modulating the reversible isomerization of first-generation DASAs were examined with dendritic OEGs carrying either methoxyl or ethoxyl terminals and DASA moieties with acceptors containing either methyl or octyl terminals. The topological effects of the dendrimer and dendronized polymers will be our focus. As shown in Figure 1, confinement from the dendronized DASAs originated through the thermal collapse of the individually dispersed dendritic OEGs (a), affording hydrophobic microenvironments via the entropy-driven, thermally induced dehydration and collapse of the dendritic OEGs. While confinement can be greatly enhanced in the copolymers through cooperative effects from neighboring dendritic OEG units along the polymer backbones, this results in the enhancement of the reversible isomerization recovery of the DASA moieties in aqueous solutions at very dilute concentrations (b).

## 2. Results and Discussion

### 2.1. Synthesis of the Dendronized DASAs and Dendronized Copolymers Carrying DASA Pendants

**Scheme 1:**
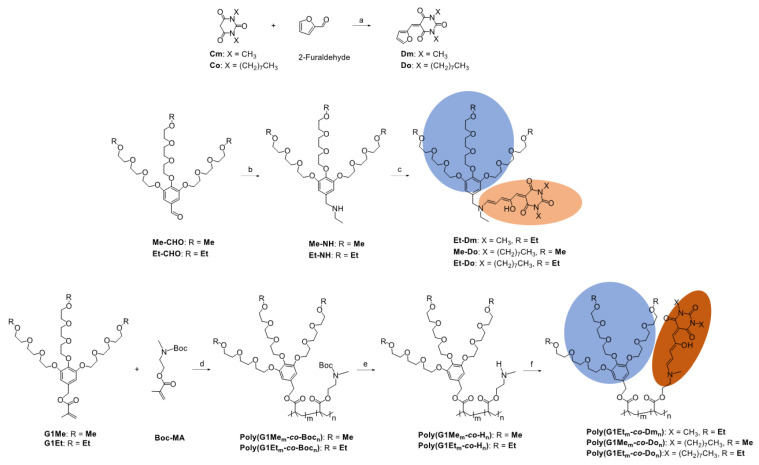
Synthesis procedures for the dendronized DASAs and the dendronized copolymers. Reagents and conditions are as follows: (**a**) H_2_O, 25 °C, 2 h (83–91%); (**b**) NaBH_4_, NaOH, Methanol, 30 °C, 3 h (40–58%); (**c**) DCM, 25 °C, 17 h (58–65%); (**d**) AIBN, DMF, 65 °C, 10 h (92–96%); (**e**) HCl, ethyl acetate, 4 h (90–95%); (**f**) DCM, 6 h (54–67%). Blue color on the dendritic OEGs indicates the hydrophilic character, and the brown colors on the DASA moieties represent the hydrophobic characters. Abbreviations are as follows: DCM = dichloromethane, AIBN = 2,2′-azobis(2-methylpropionitrile), DMF = *N*,*N*-dimethylformamide.

Three dendronized DASAs and three dendronized copolymers were designed with the aim to explore the structural effects of the confinement of encapsulated DASAs to mediate their isomerization in water. Their structural differences included the following: (1) the methoxyl or ethoxyl peripherals of the dendritic OEGs to show varied hydrophilicity, (2) the methyl or octyl for the acceptor to tailor its hydrophobic interactions with the dendritic OEGs, and (3) the spherical dendronized DASAs with low molar masses against worm-like dendronized copolymers with high molar masses, to explore the effects of molecular geometry on encapsulation. Their synthesis is outlined in Scheme 1. The dendritic **Me-NH** or **Et-NH** was cascaded with the activated furan **Dm** or **Do** to afford the dendronized DASAs **Et-Dm**, **Me-Do**, and **Et-Do** as purple oils, respectively. The copolymers carrying DASA pendants were synthesized via the attach-to route, to avoid the possible retardation of the DASA units during free radical polymerization. First, the macromonomer **G1Me** or **G1Et** was copolymerized with **Boc-MA** at designed molar ratios, affording the copolymer **Poly(G1Me_m_-*co*-Boc_n_)** or **Poly(G1Et_m_-*co*-Boc_n_)**. Due to the similar polymerization activities of these comonomers in free radical copolymerization, we assumed their copolymerization yielded random copolymers [56]. Followed by the deprotection of Boc from the random copolymers, **Poly(G1Me_m_-*co*-H_n_)** and **Poly(G1Et_m_-*co*-H_n_)** were yielded, which were reacted quantitatively with the furan derivatives **Dm** or **Do**, affording the target dendronized copolymers **Poly(G1Et_m_-*co*-Dm_n_)**, **Poly(G1Me_m_-*co*-Do_n_)**, and **Poly(G1Et_m_-*co*-Do_n_)**, respectively. Compositions for the copolymers between the dendritic moieties and DASA moieties were set to 20/1 to ensure the sufficient encapsulation of the DASA guest units from the dendritic OEG moieties [56,57]. The copolymer compositions were analyzed by ^1^H NMR spectra through proton integrations (Table S1), and the results were similar to the feed ratios of the comonomers, which are shown as footnotes for the nomenclatures of **Poly(G1Et_20_-*co*-Dm_1_)**, **Poly(G1Me_20_-*co*-Do_1_)**, and **Poly(G1Et_20_-*co*-Do_1_)**. All new compounds were characterized by ^1^H and ^13^C NMR spectroscopy (Figures S1–S10) and high-resolution mass spectrometry (Figures S11–S13) to guarantee their structures. The molar masses of the copolymers were analyzed with SEC measurements with DMF as the eluent, and they were found to be around one hundred thousand, as shown in Table S1.

### 2.2. Thermoresponsive Behavior of the Dendronized DASAs and the Dendronized Copolymers

**Figure 2:**
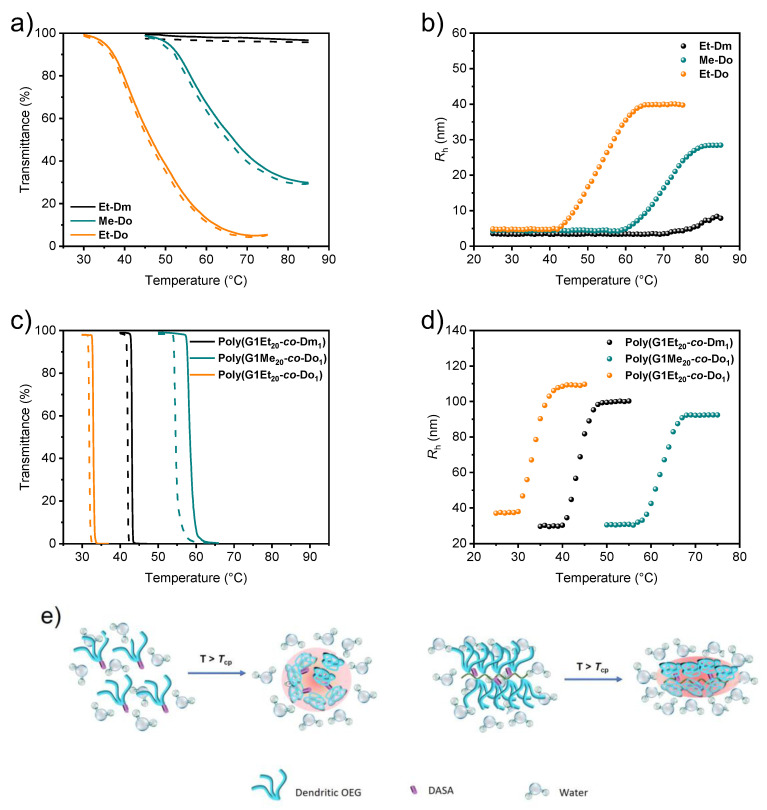
Plots of transmittance at 700 nm vs. temperature at a concentration of 1.00 mg·mL^−1^ (**a**) and plots of *R*_h_ vs. temperature at a concentration of 0.01 mg·mL^−1^ (**b**) for **Et-Dm**, **Me-Do**, and **Et-Do**. Plots of transmittance at 700 nm vs. temperature at a concentration of 1.00 mg·mL^−1^ (**c**) and plots of *R*_h_ vs. temperature in aqueous solutions at a concentration of 0.50 mg·mL^−1^ (**d**) for **Poly(G1Et_20_-*co*-Dm_1_)**, **Poly(G1Me_20_-*co*-Do_1_)**, and **Poly(G1Et_20_-*co*-Do_1_)**. Illustration for the encapsulation of DASA moieties within the dendritic macromolecules and the dendronized copolymers (**e**).

Through dendronization of DASA moieties with the dendritic OEGs, the DASA derivatives became soluble in water at room temperatures, but their solutions became turbid at elevated temperatures through entropy-driven dehydration and collapse, indicating a characteristic thermoresponsive behavior. Therefore, we tracked their thermal phase transitions by using UV/vis spectroscopy and determined their cloud point temperatures (*T*_cp_s) accordingly. Broad thermal transitions were observed for these dendronized DASAs, as shown in Figure 2a. This suggests that the dehydration and collapse of these dendronized DASAs required higher energy to counteract their strong dissolution with the increase of solution temperature. These broad transitions could also have been caused by the isomerization of the DASA moieties from a hydrophobic linear state to a hydrophilic cyclic state due to enhanced hydration during heating. For **Et-Dm,** which carried a methyl tail in the acceptor part, no obvious thermal phase transition was observed, even at a high concentration of 1.00 mmol·L^−1^. However, for **Me-Do** and **Et-Do,** which carried octyl tails in their acceptor parts, their *T*_cp_s were found to be 60.9 and 46.1 °C, respectively. The observations above indicate that the hydrophilicity change of the receptor had a key effect on the thermoresponsive behavior of the dendronized DASAs. **Et-Dm** was too hydrophilic and did not exhibit thermoresponsive behavior, but when the methyl group in the acceptor part was changed into an octyl group, as for **Et-Do**, normal thermoresponsiveness was realized with a *T*_cp_ of 46.1 °C. This indicates that the hydrophilicity of the dendritic OEGs also played an important role in mediating their thermoresponsive behavior. The methoxyl-terminated dendritic OEG in **Me-Do** contributed significantly higher hydrophilicity, and a *T*_cp_ of 14 °C higher than its ethoxyl-terminated counterpart **Et-Do** was obtained. To study the thermal aggregations of the dendronized DASAs in aqueous solutions, dynamic light scattering (DLS) measurements were performed for solutions of low concentrations to minimize multiple scattering. As depicted in Figure 2b, the hydrodynamic radius (*R*_h_) of **Et-Dm** remained constant at 8 nm when heated to 80 °C, indicating that the high hydrophilicity of this dendronized DASA prevents it from thermal aggregation. However, for the more hydrophobic **Me-Do** and **Et-Do**, aggregates were formed considerably upon heating above their *T*_cp_s, with their *R*_h_s increasing from a few nanometers at room temperature to 28 and 40 nm at higher temperatures, respectively. This indicates that thermally induced intermolecular aggregations had happened significantly for the hydrophobic dendronized DASAs.

Copolymers **Poly(G1Et_20_-*co*-Dm_1_)**, **Poly(G1Me_20_-*co*-Do_1_)**, and **Poly(G1Et_20_-*co*-Do_1_)** all exhibited characteristic thermoresponsive behavior, as shown in Figure 2c, and their *T*_cp_s were found to be 43.1, 58.4, and 32.9 °C, respectively. Copolymer **Poly(G1Et_20_-*co*-Do_1_)** showed the lowest *T*_cp_, as it carried hydrophobic ethoxyl-terminated dendritic OEGs and more hydrophobic DASA moieties with octyl tails. The difference in *T*_cp_s indicates again that hydrophilic contributions from both the peripherals of the dendritic OEGs and the receptors of DASA moieties dominate the thermoresponsive behavior of the copolymers. However, in contrast to **Et-Dm**, the copolymer **Poly(G1Et_20_-*co*-Dm_1_),** which carried the hydrophilic DASA moieties with methyl groups in its receptors, also exhibited characteristic thermoresponsive behavior with a *T*_cp_ of 43.1 °C. This was mainly due to the enhanced shielding effects to the interiors from the dendritic OEG pendants along the copolymer mainchains. The distinct thermoresponsive difference between **Et-Dm** and **Poly(G1Et_20_-*co*-Dm_1_)** implies that the DASA moieties in the dendronized copolymers were better encapsulated (and therefore, less hydrated) than those within **Et-Dm**, suggesting that the individual dendritic OEG in **Et-Dm** was not sufficient for full encapsulation of the DASA moiety, as in the case of **Poly(G1Et_20_-*co*-Dm_1_)**. The *T*_cp_s of **Poly(G1Me_20_-*co*-Do_1_)** and **Poly(G1Et_20_-*co*-Do_1_)** were similar to their corresponding homopolymers, further indicative of the efficient shielding of the dendritic OEGs for the DASA moieties in these worm-like copolymers. It is necessary to point out that the thermal phase transitions for the copolymers were much sharper than for the dendronized DASAs, which further supports that notion that the dendritic OEG pendants provided sufficient shielding to the DASA moieties, both in linear and cyclic states within the polymer interiors. As shown in Figure 2d, through increasing solution temperature from room temperature to elevated temperatures, the *R*_h_s of **Poly(G1Et_20_-*co*-Dm_1_)**, **Poly(G1Me_20_-*co*-Do_1_)**, and **Poly(G1Et_20_-*co*-Do_1_)** increased from about 30 nm to 100, 110, and 92 nm, respectively, indicating obvious thermally induced intermolecular aggregation. As illustrated in Figure 2e, worm-like dendronized copolymers provided improved confinement to the DASA moieties through cooperative interactions between neighboring dendritic OEG units via dense packing, which sharply contrasts the dendronized DASA, where only one dendritic OEG unit is available for each DASA unit. The above thermal phase transition and aggregation were followed in-situ with optical microscopy. As shown in Figure S14, no large aggregates were observed from the dendronized DASAs and the dendronized copolymers below their *T*_cp_s. However, with the increase of solution temperature above their *T*_cp_s, large spherical aggregates appeared for all the dendronized DASAs and dendronized copolymers.

### 2.3. Isomerization of the Dendronized DASAs in Water

UV/vis spectra of these dendronized DASAs in water in the dark were recorded at room temperature. The solvatochromic characteristics of DASAs allowed us to determine their maximum absorbance (λ_max_) in response to microenvironmental polarities. The more hydrophilic **Me-Do,** carrying methoxyl-terminated dendritic OEG, showed a λ_max_ of 561 nm, while the more hydrophobic **Et-Do,** carrying ethoxyl-terminated dendritic OEG, exhibited a λ_max_ of 572 nm with a bathochromic shift of 11 nm. This shift suggests that the ethoxyl-terminated dendritic OEG in **Et-Do** should have offered a notably more hydrophobic microenvironment for the DASA moieties compared to the methoxyl-terminated dendritic OEG in **Me-Do** (Figure S15a). However, **Et-Dm** with the methoxyl-terminated acceptor showed a λ_max_ of 524 nm and exhibited a large hypsochromic shift of 48 nm when compared to that of **Et-Do**. This indicates that the octyl-terminated acceptor provides hydrophobic confinement to the DASA moieties more significantly than the ethoxyl-terminated dendritic OEGs. The λ_max_ for all dendronized DASAs remained similar with the increase of their concentrations, as shown in Figure S15b–d, suggesting that there is no significant impact of concentration on the state of the DASA moieties.

**Figure 3:**
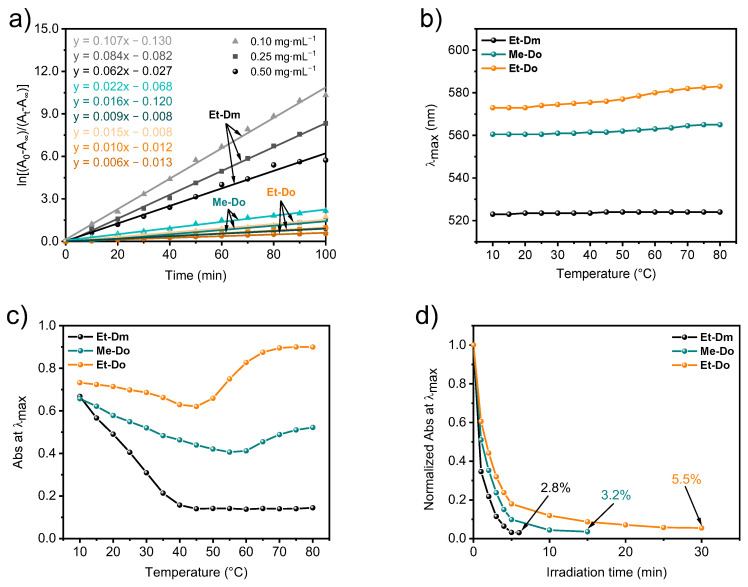
Plots of isomerization kinetics for **Et-Dm**, **Me-Do**, and **Et-Do** in water with different concentrations prior to photo-irradiation (**a**), as well as plots of λ_max_ for **Et-Dm**, **Me-Do**, and **Et-Do** vs. temperature (**b**), plots of absorbance at λ_max_ against temperature for **Et-Dm**, **Me-Do**, and **Et-Do** (**c**), and plots of absorbance at λ_max_ for **Et-Dm**, **Me-Do**, and **Et-Do** during photo-irradiation with a white LED lamp (100 W) at 25 °C (**d**). Concentration for the dendronized DASAs in (**b**–**d**) was 0.25 mmol·L^−1^.

UV/vis spectra were recorded for the dendronized DASAs in water at room temperature prior to photo-irradiation, aimed at the exploration of the isomerization of the DASA moieties within dendronized macromolecules. As shown in Figure S16, the absorbance at λ_max_ for all dendronized DASAs displayed a gradual decrease over time. This tendency indicates that the DASAs underwent spontaneous isomerization in aqueous conditions, transforming from a linear hydrophobic state to a hydrophilic cyclic state. This transformation is attributed to hydration in the highly polar aqueous medium. Nevertheless, the isomerization dynamics of these dendronized DASAs varied according to their structures. As depicted in Figure 3a, the hydrophilic **Et-Dm** demonstrated the highest isomerization kinetics from the linear to the cyclic state in an aqueous environment, while the hydrophobic compound **Et-Do** exhibited the slowest isomerization kinetics. The aforementioned findings suggest that the hydrophobic nature of dendritic macromolecules plays a crucial role in impeding the hydration of DASAs, thereby facilitating varying efficiencies in the transformation into a hydrophilic cyclic state. As the concentration of the solution increased, the absorbance at λ_max_ remained slightly elevated once isomerization reached a stable state. This observation implies that higher concentrations might have facilitated intermolecular collaboration, thereby providing enhanced protection to the DASA moieties from intense hydration. Consequently, they facilitated the preferential retention of the DASA moieties in a hydrophobic linear state.

The influence of thermally induced dehydration and the collapse of the dendronized macromolecules on the isomerization of DASAs was also monitored, and their UV/vis spectra at different temperatures were recorded (Figure S17). Figure 3b,c illustrate the transitions of λ_max_ and the corresponding absorbance at λ_max_ for various dendronized DASAs during the thermal phase transitions. Increasing solution temperature from 10 to 80 °C for **Et-Dm** and **Me-Do**, which are known for their enhanced hydrophilicity, resulted in a bathochromic shift of λ_max_ from 523 to 524 nm and 561 to 565 nm, respectively. This phenomenon indicates that the thermal dehydration of the dendritic OEGs facilitates the formation of thermally aggregated domains, thereby creating a more hydrophobic microenvironment for the DASA moieties. The absorbance at λ_max_ underwent a significant reduction during the phase transitions, suggesting the isomerization of the majority of DASA moieties into the cyclic state as a consequence of increased hydration due to rising temperature. The absorption spectrum of the more hydrophobic **Et-Do** showed a bathochromic shift of its λ_max_ from 573 to 583 nm with the increase in solution temperature from 10 to 80 °C, demonstrating its solvatochromic properties. Surprisingly, the absorbance at λ_max_ showed a remarkable increase along with the temperature. This observation implies that the thermal dehydration and subsequent collapse of the ethoxyl-terminated dendritic OEGs led to the formation of more hydrophobic domains. These domains, facilitated by hydrophobic interactions originating from the octyl-terminated acceptor, resulted in a significantly enhanced hydrophobic confinement of the DASA moieties. Consequently, this confinement not only impedes the isomerization of the moieties into the hydrophilic cyclic state but also induces the cyclic isomer to transfer into the hydrophobic linear state within the aqueous environment. The above results suggest that the hydrophobicity of the microenvironment formed by dehydrated and collapsed dendritic OEGs, along with the cooperative interactions between the crowded OEG chains and the DASA moiety, can modulate the isomerization of DASAs within the matrix of dendritic OEGs. This finding implies that regulating interactions with dendritic OEGs can efficiently control the isomerization of DASA in water.

The isomerization of the dendronized DASAs in water below their phase transition temperature was further examined through photo-irradiation, and the results are shown in Figure 3d. The absorbance at λ_max_ for all dendronized DASAs was reduced greatly through photo-irradiation with visible light. This indicates efficient isomerization of the DASA moieties from the linear to the cyclic state, modulated by photo-irradiation. Among all dendronized DASAs examined, **Et-Dm** exhibited the fastest photoisomerization from a linear to a cyclic state, similar to their spontaneous isomerization in water under dark. When they reached photo-stationary states, absorbances at λ_max_ for **Et-Dm**, **Me-Do**, and **Et-Do** became 2.8%, 3.2% and 5.5% of their initial values, respectively, when the solution concentrations were set to be 0.25 mg·mL^−1^. These results indicate that the dendritic macromolecules are supportive for isomerization of the DASA moieties from the hydrophobic linear state into the hydrophilic cyclic state when they are more hydrophilic.

**Figure 4:**
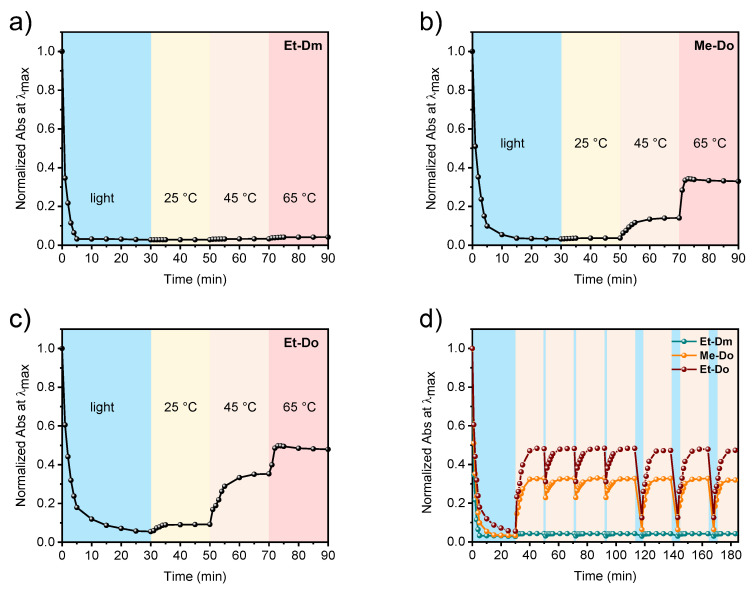
Plots of normalized absorbance at λ_max_ for **Et-Dm** (**a**), **Me-Do** (**b**), and **Et-Do** (**c**) against time after photo-irradiation at room temperature and followed by annealing at different temperature (in dark), as well as plots of absorbance at λ_max_ for **Et-Dm**, **Me-Do**, and **Et-Do** through alternative photo-irradiation (for 1 min) and thermal annealing at 65 °C (for 5 min) (**d**). Sample concentration = 0.25 mg·mL^−1^.

The spontaneous isomerization recovery for the DASA moieties from **Et-Do** at elevated temperatures, as shown in Figure 3c, demonstrates that the isomerization recovery of the DASA moieties in aqueous conditions from the hydrophilic cyclic isomer into the hydrophobic linear state can be modulated. This is due to the enhanced interactions from the DASA moieties carrying a hydrophobic receptor with the dehydrated hydrophobic dendritic OEG domains, which provide sufficient driving force. This intriguing feature encouraged us to investigate the effects of thermal dehydration on the isomerization recovery of the dendronized DASAs in water after photo-irradiation. Their UV/vis spectra in water after photoirradiation were recorded through annealing at elevated temperatures for different durations. The absorbance at λ_max_ for the hydrophilic **Et-Dm** decreased with the elongation of photo-irradiation time, which remained weak after annealing at different temperatures (Figure 4a and Figure S18), which suggests that the cyclic isomer from the hydrophilic **Et-Dm** was stable enough in the aqueous phase. However, absorbance at λ_max_ for the more hydrophobic **Me-Do** decreased with the elongation of photo-irradiation time, which was partially recovered at different temperatures in the dark (Figure 4b and Figure S19). The absorbance remained weak after the solution was kept for 20 min at 25 °C (below its *T*_cp_), which then increased to 14.0% at 45 °C (slightly above its *T*_cp_) and 32.9% at 65 °C (much higher than its *T*_cp_). Accordingly, the isomerization recovery of DASA moieties from **Me-Do** at 45 °C and 65 °C was found to be 14.0% and 32.9%, respectively. This indicates that the octyl-terminated acceptor in the DASAs provides hydrophobicity to support their isomerization recovery from a hydrophilic cyclic to a hydrophobic linear state. Moreover, this recovery can be significantly enhanced through the thermally mediated collapse and aggregation of the OEG moieties. The isomerization recovery for more highly hydrophobic **Et-Do** is even better at elevated temperatures. The efficient isomerization of the DASA moieties into the hydrophilic cyclic state through photo-irradiation was observed (Figure 4c and Figure S20), which was recovered through annealing at different temperatures. Overall, isomerization recovery for the DASA moieties in **Et-Do** at 25 °C, 45 °C, and 65 °C was found to be 9.2%, 35.3%, and 47.9%, respectively. This thermally annealing-mediated isomerization recovery was repeated several times for all dendronized DASAs to demonstrate its high reversibility and reliability. As shown in Figure 4d, the isomerization recovery showed excellent reversibility for all dendronized DASAs during multiple photo-irradiations and annealings at elevated temperatures.

Since photo-irradiation can induce the isomerization of the DASA moieties into a more hydrophilic cyclic state, it may influence thermal aggregation of the dendronized DASAs. Therefore, possible effects of photo-irradiation on their thermal aggregations were monitored by DLS measurements, and the results are shown in Figure S21a. **Et-Dm** showed a similar *R*_h_ from room temperature to elevated temperatures after photo-irradiation, suggesting that **Et-Dm** is too hydrophilic to exhibit typical thermoresponsive behavior. Alternatively, the more hydrophobic **Me-Do** and **Et-Do** showed thermal aggregation behavior. After photo-irradiation, their *R*_h_s increased from a few nanometers at room temperature to 22 and 33 nm at elevated temperatures, respectively, indicating that thermally induced aggregation remained when these dendronized DASAs were in the more hydrophilic cyclic state. Through alternative reversible isomerization, **Me-Do** and **Et-Do** showed similar thermal aggregation behavior, as shown in Figure S21b. This suggests that photo-isomerization can be highly reversible through thermal aggregation. Increasing the concentration helped the isomerization recovery, as shown in Figure S22. For these dendronized DASAs, the isomerization recovery at elevated temperatures was enhanced significantly with the increase of concentrations. This concentration-enhanced recovery was especially pronounced for **Et-Do**, whose isomerization recovery was increased from 11.6% to 81.8% when its concentration was increased from 0.10 to 0.50 mg·mL^−1^ (Figure S22c).

### 2.4. Reversible Isomerization of the DASAs Through Enhanced Confinement from the Dendronized Copolymers

**Figure 5:**
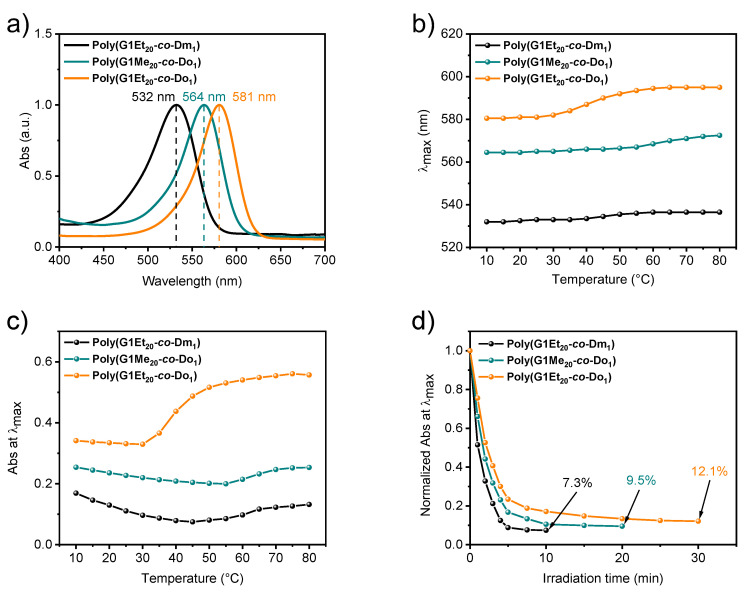
UV/vis spectra of **Poly(G1Et_20_-*co*-Dm_1_)**, **Poly(G1Me_20_-*co*-Do_1_)**, and **Poly(G1Et_20_-*co*-Do_1_)** in aqueous solutions (**a**), as well as plots of λ_max_ for **Poly(G1Et_20_-*co*-Dm_1_)**, **Poly(G1Me_20_-*co*-Do_1_)**, and **Poly(G1Et_20_-*co*-Do_1_)** against temperature (**b**), plots of absorbance at λ_max_ against temperature for **Poly(G1Et_20_-*co*-Dm_1_)**, **Poly(G1Me_20_-*co*-Do_1_)**, and **Poly(G1Et_20_-*co*-Do_1_)** (**c**), and plots of absorbance at λ_max_ for **Poly(G1Et_20_-*co*-Dm_1_)**, **Poly(G1Me_20_-*co*-Do_1_)**, and **Poly(G1Et_20_-*co*-Do_1_)** during photo-irradiation with a white LED lamp (100 W) at 25 °C (**d**). Concentration = 0.25 mg·mL^−1^.

In order to explore the effects of molecular geometry on the encapsulation and isomerization of the DASAs, dendronized polymethacrylates carrying DASA pendants were examined. UV/vis spectra of aqueous solutions of **Poly(G1Et_20_-*co*-Dm_1_)**, **Poly(G1Me_20_-*co*-Do_1_)**, and **Poly(G1Et_20_-*co*-Do_1_)** were recorded to investigate their isomerization recovery. As shown in Figure 5a, the λ_max_ corresponding to the DASA moieties from **Poly(G1Et_20_-*co*-Dm_1_)**, **Poly(G1Me_20_-*co*-Do_1_)**, and **Poly(G1Et_20_-*co*-Do_1_)** red-shifted from 524 nm to 532, 564, and 581 nm, respectively. This indicates that the DASA moieties were encapsulated to a certain degree, and the dendritic OEGs along the polymer backbones provided them with an enhanced hydrophobic microenvironment. However, the λ_max_ for all dendritic copolymers remained unchanged with the increase of their concentrations (Figure S23), sharp contrasting the case of the dendronized DASAs. This indicates that the DASA moieties were efficiently encapsulated by the dendritic OEGs along the polymer backbone. To investigate the possible effects of the thermal dehydration and collapse of the dendritic OEGs on solvation of the DASA moieties, UV/vis spectra of the dendronized copolymers across their thermal phase transitions were recorded (Figure S24). The corresponding transitions of the λ_max_ and the absorbance at λ_max_ for different dendronized copolymers are plotted in Figure 5b,c. For the more hydrophilic copolymers **Poly(G1Et_20_-*co*-Dm_1_)** and **Poly(G1Me_20_-*co*-Do_1_)**, the increase of solution temperatures from 10 to 80 °C led to slightly bathochromic shifts of the λ_max_ from 532 to 537 nm and 564 to 573 nm, respectively. This indicates that the dehydrated and collapsed domains from the dendritic OEG provide a more hydrophobic microenvironment to encapsulate the DASA moieties. Comparing these bathochromically shifted λ_max_s with those corresponding to the dendronized DASAs, copolymers afforded the DASA moieties much more enhanced hydrophobic microenvironments through cooperative interactions between neighboring dendritic OEGs. However, the reduced absorbance at λ_max_ from the copolymers indicates that most of the DASA moieties should have been isomerized into the cyclic state through heating in aqueous solutions. Alternatively, the λ_max_ for the more hydrophobic **Poly(G1Et_20_-*co*-Do_1_)** obviously red-shifted from 581 to 595 nm when the solution temperature increased from 10 to 80 °C, and, simultaneously, the absorbance at λ_max_ obviously increased. The above observations indicate that the isomerization of DASAs is related to both the hydrophobicity of the microenvironment created from the dehydrated and collapsed dendritic OEGs along the polymer backbones, together with the cooperative interactions of the dehydrated and crowded OEG chains with the DASA moieties. Therefore, the isomerization of DASAs in water can be modulated simply through manipulating their interactions with the dendritic OEGs within the matrices of the copolymers.

This study initially focused on investigating the photo-irradiation-mediated isomerization of DASAs within dendronized copolymers in water at room temperature (below their thermal phase transition temperature). As shown in Figure 5d, a significant decrease in absorbance at λ_max_ was observed for all dendronized copolymers within a few minutes of exposure to visible light. This indicates a notable accelerated isomerization of DASA moieties from a linear to a cyclic state within these dendronized copolymers through photo-irradiation. Upon reaching the photo-stationary states, the absorbances at λ_max_ for **Poly(G1Et_20_-*co*-Dm_1_)**, **Poly(G1Me_20_-*co*-Do_1_)**, and **Poly(G1Et_20_-*co*-Do_1_)** at a concentration of 0.25 mg·mL^−1^ reached 7.3%, 9.5%, and 12.1% of their initial values, respectively. This suggests that the more hydrophilic dendronized copolymers may slightly facilitate the photoisomerization process.

**Figure 6:**
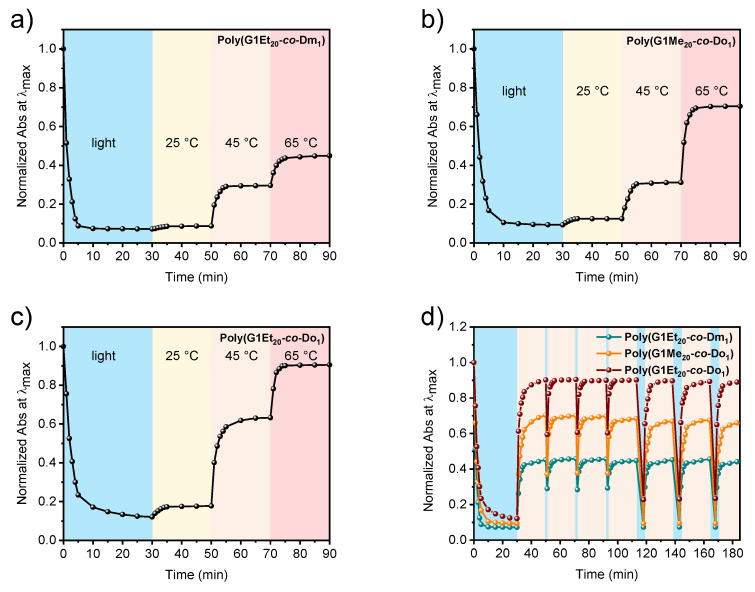
Plots of normalized absorbance at λ_max_ for **Poly(G1Et_20_-*co*-Dm_1_)** (**a**), **Poly(G1Me_20_-*co*-Do_1_)** (**b**), and **Poly(G1Et_20_-*co*-Do_1_)** (**c**) after photo-irradiation for different times (at room temperatures) and followed by annealing at different temperatures (in dark), as well as plots of absorbance at λ_max_ for **Poly(G1Et_20_-*co*-Dm_1_)**, **Poly(G1Me_20_-*co*-Do_1_)**, and **Poly(G1Et_20_-*co*-Do_1_)** after alternative photo-irradiation (for 1 min) and thermal annealing at 65 °C (for 5 min). (**d**) Polymer concentration = 0.25 mg·mL^−1^.

The reversible isomerization of the DASA moieties in **Poly(G1Et_20_-*co*-Dm_1_)**, **Poly(G1Me_20_-*co*-Do_1_)**, and **Poly(G1Et_20_-*co*-Do_1_)** was subjected to further examination through photo-irradiation. The analysis depicted in Figure 6a and Figure S25 reveals a reduction in the absorbance at λ_max_ for **Poly(G1Et_20_-*co*-Dm_1_)** after photo-irradiation, with partial recovery through annealing at various temperatures in the dark. The absorbance when maintained at 25 °C (below its *T*_cp_) remained weak after a 20 min duration. Notably, this weak absorbance could be greatly heightened to 29.6% at 45 °C (slightly exceeding its *T*_cp_) and to 44.9% at 65 °C (considerably transcending its *T*_cp_). Consequently, the isomerization recovery for DASA moieties in **Poly(G1Et_20_-*co*-Dm_1_)** from the hydrophilic cyclic state to the hydrophobic linear state at 45 °C and 65 °C was documented at 29.6% and 44.9%, respectively. The isomerization recovery of the DASA moieties in the more hydrophobic **Poly(G1Me_20_-*co*-Do_1_)** and **Poly(G1Et_20_-*co*-Do_1_)** demonstrates enhanced efficiency at elevated temperatures, as shown in Figure 6b,c (for UV/vis spectra, refer to Figures S26 and S27). Upon photo-irradiation, the DASA moieties underwent rapid isomerization, maintaining the hydrophilic cyclic state, which could be restored to the hydrophobic linear state under dark conditions through annealing at varying temperatures. The isomerization recovery percentages for the more hydrophilic **Poly(G1Me_20_-*co*-Do_1_)** at 25 °C, 45 °C, and 65 °C were 12.5%, 31.2%, and 70.4%, respectively. For the more hydrophobic **Poly(G1Et_20_-*co*-Do_1_)**, the corresponding percentages were 17.8%, 63.3%, and 90.2%. The above observations indicate that the isomerization recovery of the DASAs in the worm-like copolymers is much more enhanced when compared to that in the spherical dendronized DASAs. This further demonstrates that the cooperative effects from multiple dendritic OEGs within the dendronized copolymers, especially when polymer chains are densely packed at elevated temperatures through thermal collapse, play an important role in the confinement of the DASA moieties to enhance their isomerization back into the hydrophobic linear state in the aqueous phase. The process of isomerization recovery via thermal annealing was conducted multiple times for all dendronized copolymers, exhibiting notable reversibility and reliability. As depicted in Figure 6d, the isomerization recovery exhibited exceptional reversibility when subjected to alternating photo-irradiation and heating.

## 3. Experimental Section

### 3.1. Materials

The compounds **Cm** [36], **Co** [36], **Dm** [36], **Do** [36], **Me-CHO** [50], **Et-CHO** [50], **Me-NH [50]**, **Et-NH [50]**, **Boc-MA [55]**, **G1Me** [50], and **G1Et** [50] were prepared according to procedures found in the literature. DCM, THF, and methanol were purchased from Sinopharm Chemical Reagent Co., Ltd. (Shanghai, China). Dry *N,N*-dimethylformamide (DMF), CaH_2_, NaH, LiAlH_4_ and potassium carbonate were purchased from Sigma-Aldrich Chemical Co., Ltd. (Shanghai, China). AIBN was purchased from Aladdin Bio-Chem Technology Co., Ltd. (Shanghai, China). DCM was dried over CaH_2_. THF was predried over NaH and then refluxed over LiAlH_4_ before use. Potassium carbonate was predried at 100.0 °C in vacuo overnight. Dry *N,N*-dimethylformamide (DMF) was purchased from Acros and was used as received. AIBN was recrystallized twice in methanol. Other reagents and solvents were of reagent grade and used without further purification. All reactions were run under nitrogen atmosphere.

### 3.2. Instrumentation and Measurements

^1^H and ^13^C NMR spectra were recorded on a Bruker Advance Neo 500 (^1^H: 500 MHz; ^13^C: 125 MHz) spectrometer. DLS experiments were performed on an ALV/CGS-3 instrument (scattering angle, 90°). Size exclusion chromatography (SEC) measurements were carried out on a Waters GPC e2695 instrument at 45 °C with three columns set (Styragel HR3  +  HR4  +  HR5), equipped with a refractive index detector (Waters 2414). DMF (containing 1 mg∙mL^−1^ of LiBr) was used as eluent. The calibration was performed with poly(methyl methacrylate) standards in the range of M*_p_* =  2540 to 936,000 (Polymer Standards Service-USA, Inc., Santa Clara, CA, USA). UV/vis spectra were recorded on a PE UV/vis spectrometer Lambda 35, equipped with a thermostatically regulated bath. Absorptions of the solutions at λ = 700 nm were recorded every five seconds. The cloud point temperature (*T*_cp_) was determined as the point when transmittance at λ = 700 nm reached 50% of its initial value. Visible light photo-irradiation was performed at 25 °C by a white LED lamp (Model HL-L20 from RESTART Corp., Shenzhen, China) with an output power of 100 W, and the distance between sample and light source was set to 25 cm.

### 3.3. Synthesis

#### 3.3.1. 5-((2*Z*,4*E*)-5-(Ethyl(3,4,5-tris(2-(2-(2-ethoxyethoxy)ethoxy) ethoxy) benzyl) amino) -2-hydroxypenta-2,4-dien-1-ylidene)-1,3-dimethylpyrimidine-2,4,6(1*H*,3*H*,5*H*)-trione (Et-Dm)

Compound **Et-NH** (663.9 mg, 1 mmol) and **Dm** (234.2 mg, 1 mmol) were dissolved in DCM (8 mL) and stirred at 40 °C in the dark for 17 h. After removal of organic solvent by vacuum distillation, the purple product was repeatedly washed with n-hexane to obtain a dark-purple oil, **Et-Dm** (321.5 mg, 35.8%). ^1^H NMR (500 MHz, CDCl_3_): δ = 1.17–1.30 (m, 12H, CH_3_), 3.48 (m, 6H, CH_2_), 3.52–4.00 (m, 38H, CH_2_), 4.14 (br, 6H, CH_2_), 4.50 (d, 2H, CH_2_), 6.07–6.18 (m, 1H, CH), 6.45 (t, 2H, CH), 6.79 (br, 1H, CH), 7.21 (s, 1H, CH), 7.30–7.36 (m, 1H, CH). ^13^C NMR (125 MHz, CDCl_3_): δ = 166.58, 159.62, 150.15, 146.84, 138.55, 136.43, 131.29, 129.86, 101.48, 87.33, 72.05, 70.78, 70.66, 70.63, 70.54, 70.50, 70.43, 70.32, 70.12, 69.80, 69.69, 69.54, 69.44, 69.17, 66.68, 66.64, 31.93, 29.70, 29.33, 28.48, 28.30, 22.69, 22.60, 15.15, 15.07, 15.04, 14.13. HR-MS (ESI): *m*/*z* calcd for C_44_H_71_N_3_O_16_ [M + H]^+^, 898.4907; found, 898.4896.

#### 3.3.2. 5-((2*Z*,4*E*)-5-(Ethyl(3,4,5-tris(2-(2-(2-methoxyethoxy)ethoxy)ethoxy) benzyl) amino)-2-hydroxypenta-2,4-dien-1-ylidene)-1,3-dioctylpyrimidine-2,4,6(1*H*,3*H*, 5*H*)-trione (Me-Do)

With a similar procedure as that for **Et-Dm,** using **Me-NH** (621.8 mg, 1 mmol) and **Do** (430.6 mg, 1 mmol), **Me-Do** was obtained as a dark-purple oil (678.7 mg, 64.5%). ^1^H NMR (500 MHz, CDCl_3_): δ = 0.88 (s, 2H, CH), 1.25–1.30 (t, 9H, CH_3_), 2.01 (t, 3H, CH_3_), 3.36–3.37 (s, 9H, CH_3_), 3.53–3.84 (m, 36H, CH_2_), 4.13–4.14 (s, 6H, CH_2_), 6.44 (s, 2H, CH), 7.23 (s, 1H, CH). ^13^C NMR (125 MHz, CDCl_3_): δ = 166.61, 159.75, 152.72, 149.30, 148.20, 140.51, 136.75, 130.70, 128.93, 102.09, 87.21, 72.28, 71.95, 71.93, 71.88, 70.78, 70.73, 70.68, 70.59, 70.53, 70.53, 69.70, 68.83, 59.03, 59.01, 31.83, 29.71, 29.35, 29.25, 28.21, 27.02, 22.65, 14.11. HR-MS (ESI): *m*/*z* calcd for C_55_H_93_N_3_O_16_ [M + H]^+^, 1053.6448; found, 1053.6613.

#### 3.3.3. 5-((2*Z*,4*E*)-5-(Ethyl(3,4,5-tris(2-(2-(2-ethoxyethoxy)ethoxy)ethoxy) benzyl) amino) -2-hydroxypenta-2,4-dien-1-ylidene)-1,3-dioctylpyrimidine-2,4,6(1*H*,3*H*, 5*H*)-trione (Et-Do)

With a similar procedure as that for **Et-Dm**, using **Et-NH** (663.9 mg, 1 mmol) and **Do** (430.6 mg, 1 mmol), **Et-Do** was obtained as a dark-purple oil (681.9 mg, 62.3%). ^1^H NMR (500 MHz, CDCl_3_): δ = 0.86–0.88 (t, 6H, CH_3_), 1.18–1.21 (m, 12H, CH_3_), 1.25–1.33 (m, 32H, CH_2_, CH_3_), 1.60–1.62 (d, 8H, CH_2_), 3.48 (m, 2H, CH_2_), 3.50–3.92 (m, 36H, CH_2_), 4.12–4.15 (s, 6H, CH_2_), 6.44 (s, 2H, CH), 7.23 (s, 1H, CH). ^13^C NMR (125 MHz, CDCl_3_): δ = 166.43, 160.33, 152.79, 149.73, 147.68, 138.32, 136.26, 131.53, 129.48, 101.37, 86.14, 72.34, 70.80, 70.66, 70.63, 70.52, 69.83, 69.81, 69.78, 68.91, 66.63, 59.68, 48.97, 46.16, 31.83, 29.71, 29.34, 29.25, 28.20, 27.02, 22.65, 22.62, 15.16, 15.14, 14.11. HR-MS (ESI): *m*/*z* calcd for C_58_H_99_N_3_O_16_ [M + H]^+^, 1095.7098; found, 1095.7084.

#### 3.3.4. Poly(3,4,5-tris(2-(2-(2-ethoxyethoxy) ethoxy)ethoxy) benzyl meth-acrylate)-co-(2-((tert-butoxycarbonyl)(methyl)amino)ethyl methacrylate) [Poly(G1Et_20_-co-Boc_1_)]

Monomer **G1Et** (422.8 mg, 20 mmol), **Boc-MA** (6.6 mg, 1 mmol), and AIBN (2.1 mg, 13 μmol) were mixed in DMF (750 μL) and subjected to vacuuming for 30 min, then they were stirred at 65 °C for 18 h in the dark under the protection of nitrogen. After removal of organic solvent by vacuum, the crude product was purified through column chromatography on silica gel with DCM as eluent to afford **Poly(G1Et_20_-*co*-Boc_1_)** as a colorless oil (412.5 mg, 96.1%). ^1^H NMR (500 MHz, CDCl_3_): δ = 0.92–1.05 (m, 3H, CH_3_), 1.16–1.28 (m, 9H, CH3), 2.89 (s, 3H, CH_3_), 3.48–3.84 (m, 36H, CH_2_), 4.03–4.32 (m, 10H, CH_2_), 6.29–6.30 (d, 1H, CH), 6.79 (s, 2H, CH), 7.44 (m, 1H, CH), 7.56 (d, 1H, CH).

#### 3.3.5. Poly(3,4,5-tris(2-(2-(2-methoxyethoxy)ethoxy)ethoxy)benzyl meth-acrylate)-co-(2-((tert-butoxycarbonyl)(methyl)amino)ethyl methacrylate) [Poly(G1Me_20_-co-Boc_1_)]

With a similar procedure as that for **Poly(G1Et_20_-*co*-Boc_1_),** using **G1Me** (397.3 mg, 20 mmol), **Boc-MA** (6.6 mg, 1 mmol), and AIBN (2.1 mg, 13 μmol), **Poly(G1Me_20_-*co*-Boc_1_)** was obtained as a colorless oil (386.2 mg, 95.3%). ^1^H NMR (500 MHz, CDCl_3_): δ = 0.81–1.07 (m, 3H, CH_3_), 1.27 (m, 9H, CH_3_), 3.34–3.76 (m, 36H, CH_2_), 4.08 (m, 10H, CH_2_), 4.79 (d, 2H, CH_2_), 6.51 (s, 2H, CH).

#### 3.3.6. Poly(3,4,5-tris(2-(2-(2-ethoxyethoxy)ethoxy)ethoxy)benzyl meth-acrylate)-co-(2-(dimethylamino)ethyl methacrylate) [Poly(G1Et_20_-co-H_1_)]

Copolymer **Poly(G1Et_20_-*co*-Boc_1_)** (180.0 mg, 1 mmol) was dissolved in ethyl acetate solution (300 μL, 40 mmol) with HCl (146 mg, 4 mmol) and the mixture was stirred in the dark at room temperature for 4 h. After removal of organic solvent by vacuum, the crude product was washed with saturated NaHCO_3_ aqueous solution to afford **Poly(G1Et_20_-*co*-H_1_)** as a colorless oil (100.6 mg, 55.9%). ^1^H NMR (500 MHz, CDCl_3_): δ = 0.87–0.96 (m, 3H, CH_3_), 1.15–1.37 (m, 15H, CH_3_), 1.68 (s, 3H, CH_3_), 3.45–3.81 (m, 38H, CH_2_), 4.12–4.31 (m, 10H, CH_2_), 6.75 (s, 2H, CH).

#### 3.3.7. Poly(3,4,5-tris(2-(2-(2-methoxyethoxy)ethoxy)ethoxy)benzyl meth-acrylate)-co-(2-(dimethylamino)ethyl methacrylate) [Poly(G1Me_20_-co-H_1_)]

With a similar procedure as that for **Poly(G1Et_20_-*co*-H_1_),** using **Poly(G1Me_20_-*co*-Boc_1_)** (169.2 mg, 1 mmol), **Poly(G1Me_20_-*co*-H_1_)** was obtained as a colorless oil (90.8 mg, 53.7%). ^1^H NMR (500 MHz, CDCl_3_): δ = 0.90 (m, 3H, CH_3_), 1.07–1.36 (m, 15H, CH_3_), 1.74 (s, 3H, CH_3_), 3.44–3.85 (m, 38H, CH_2_), 4.13–4.37 (m, 10H, CH_2_), 4.91 (d, 2H, CH_2_), 6.55 (s, 2H, CH). 

#### 3.3.8. Poly(3,4,5-tris(2-(2-(2-ethoxyethoxy)ethoxy)ethoxy)benzyl meth-acrylate)-co-(2-(((1*E*,3*Z*)-5-(1,3-dimethyl-2,4,6-trioxotetrahydropyrimidin-5(2*H*)-ylidene)-4-hydroxy-penta-1,3-dien-1-yl)(methyl)amino)ethyl methacrylate) [Poly(G1Et_20_-co-Dm_1_)]

Copolymer **Poly(G1Et_20_-*co*-H_1_)** (284.8 mg, 20 μmol) and **Dm** (1.2 mg, 5 μmol) were mixed in DCM (2 mL) and stirred at room temperature for 6 h in the dark. After removal of organic solvent by vacuum distillation, the crude product was repeatedly washed with n-hexane to obtain a dark-purple oil of **Poly(G1Et_20_-*co*-Dm_1_)** (154.9 mg, 54.2%). ^1^H NMR (500 MHz, CDCl_3_): δ = 1.20 (m, 11H, CH_3_), 3.46–3.59 (m, 14H, CH_2_), 3.59–3.67 (m, 15H, CH_2_), 3.70 (s, 5H, CH_2_), 3.79 (d, 6H, CH_2_), 4.10 (s, 3H, CH_2_), 6.53 (s, 1H, CH). ^13^C NMR (125 MHz, CDCl_3_): δ = 176.01, 166.08, 160.17, 152.63, 150.53, 148.78, 139.24, 137.85, 130.13, 127.98, 105.51, 101.98, 87.66, 72.28, 70.70, 70.61, 70.56, 70.44, 69.82, 69.80, 69.66, 68.78, 66.56, 66.51, 62.17, 61.94, 58.83, 43.09, 29.76, 24.49, 15.21.

#### 3.3.9. Poly(3,4,5-tris(2-(2-(2-methoxyethoxy)ethoxy)ethoxy)benzyl meth-acrylate)-co-(2-(((1*E*,3*Z*)-5-(1,3-dioctyl-2,4,6-trioxotetrahydropyrimidin-5(2*H*)-ylidene)-4-hydroxypenta -1,3-dien-1-yl)(methyl)amino)ethyl methacrylate) [Poly(G1Me_20_-co-Do_1_)]

With a similar procedure as that for **Poly(G1Et_20_-*co*-Dm_1_),** using **Poly(G1Me_20_-*co*-H_1_)** (267.7 mg, 20 μmol) and **Do** (2.2 mg, 5 μmol), **Poly(G1Me_20_-*co*-Do_1_)** was obtained as a dark-purple oil (180.3 mg, 66.8%). ^1^H NMR (500 MHz, CDCl_3_): δ = 0.77 (s, 1H, CH_3_), 3.34 (d, 20H, CH_2_), 3.46–3.55 (m, 16H, CH_2_), 3.58 (s, 15H, CH_2_), 3.62 (s, 12H, CH_2_), 3.67 (d, 16H, CH_2_), 3.75 (s, 8H, CH_2_), 4.05 (d, 10H, CH_2_), 4.78 (s, 1H, CH_2_), 6.50 (s, 2H, CH). ^13^C NMR (125 MHz, CDCl_3_): δ = 175.17, 166.37, 160.67, 152.60, 150.33, 147.96, 137.88, 131.67, 128.83, 107.00, 101.36, 86.65, 72.28, 71.92, 71.88, 70.67, 70.61, 70.56, 70.46, 70.43, 69.65, 68.76, 62.21, 58.96, 44.47, 36.13, 31.83, 29.70, 25.44, 22.65, 14.13.

#### 3.3.10. Poly(3,4,5-tris(2-(2-(2-ethoxyethoxy)ethoxy)ethoxy)benzyl meth-acrylate)-co-(2-(((1*E*,3*Z*)-5-(1,3-dioctyl-2,4,6-trioxotetrahydropyrimidin-5(2*H*)-ylidene)-4-hydroxy-penta-1,3-dien-1-yl)(methyl)amino)ethyl methacrylate) [Poly(G1Et_20_-co-Do_1_)]

With a similar procedure as that for **Poly(G1Et_20_-*co*-Dm_1_),** using **Poly(G1Et_20_-*co*-H_1_)** (284.8 mg, 20 μmol) and **Do** (2.2 mg, 5 μmol), **Poly(G1Et_20_-*co*-Do_1_)** was obtained as a dark-purple oil (173.1 mg, 60.3%).^1^H NMR (500 MHz, CDCl_3_): δ = 0.77–0.83 (m, 1H, CH_3_), 1.17 (d, 11H, CH_3_), 3.48 (s, 5H, CH_2_), 3.52 (s, 7H, CH_2_), 3.58 (s, 10H, CH_2_), 3.65 (t, 18H, CH_2_), 3.74 (s, 5H, CH_2_), 4.03–4.07 (s, 3H, CH_2_), 6.49 (s, 1H, CH). ^13^C NMR (125 MHz, CDCl_3_): δ = 175.09, 166.23, 160.30, 152.62, 149.97, 148.30, 139.43, 137.89, 130.63, 128.65, 106.97, 101.25, 87.72, 72.28, 70.68, 70.60, 70.55, 70.43, 69.82, 69.79, 69.65, 68.77, 66.56, 66.51, 61.13, 58.28, 45.13, 36.31, 32.49, 29.43, 25.43, 22.64, 15.22, 14.34.

## 4. Conclusions

Combining DASAs within either spherical dendritic topologies or worm-like dendronized polymers, we have demonstrated that dendritic architecture exhibits unique confinement to the encapsulated DASA moieties through crowding and mediates their reversible isomerization in water. Originating from the dendritic OEGs, these dendritic macromolecules exhibit unprecedented thermoresponsive behavior to form hydrophobic microdomains through thermal dehydration and collapse. Although the dendronized DASAs show the ability to mediate to some extent the encapsulation of the DASA moieties, worm-like dendronized polymers provide more efficient shielding to the guest moieties through cooperation between neighboring dendritic OEGs, modulating their thermoresponsive behavior, which is dominated by the dendritic OEG pendants. Thermal aggregation-formed hydrophobic domains from dendronized copolymers exhibit much more enhanced confinement of the DASA moieties’ hydration, which can mediate their isomerization recovery in water, allowing for the charged (hydrophilic) DASA isomer to transform into the non-charged (hydrophobic) linear state by up to 90% at dilute concentrations. This isomerization recovery can be repeated multiple times through alternating photo-irradiation and thermal annealing at elevated temperatures, which exhibited excellent fatigue resistance. The principle developed in this report should also be appliable to modulate reversible isomerization of other hydrophobic dyes in water, and therefore, may have opened a convenient pathway for developing photochromic materials for applications in aqueous environments.

## Data Availability

Data are contained within the article and Supplementary Materials.

## Supplementary Materials

The following supporting information can be downloaded at: https://www.mdpi.com/article/10.3390/molecules29215055/s1. Figures S1–S10: NMR spectra of dendronized DASAs and the dendronized copolymers; Figures S11–S13: HR-MS spectra of dendronized DASAs; Table S1: Conditions for and results from the copolymerization of **G1Me** or **G1Et** with **Dm** or **Do**; Figure S14: Optical micrographs of the aqueous solutions from dendronized DASAs and the corresponding dendronized copolymethacrylates below and above their *T*_cp_s; Figures S15–S22: UV/vis spectra of dendronized DASAs in aqueous solutions under various conditions; Figure S23–S27: UV/vis spectra of dendronized copolymers in aqueous solutions under various conditions.
