# Supplementary material for: Crowding for Confinement: Reversible Isomerization of First-Generation Donor-Acceptor Stenhouse Adduct Derivatives in Water Modulated by Thermoresponsive Dendritic Macromolecules
Source: Molecules. 2024 Oct 26;29(21):5055. doi: 10.3390/molecules29215055 (PMC11547267; doi:10.3390/molecules29215055)
Supplement: Supplementary file 1 [file molecules-29-05055-s001.zip › molecules-3191590-supplementary.pdf]

Supplementary Information

Crowding for Confinement: Reversible Isomerization of First-Generation Donor-Acceptor Stenhouse Adduct Derivatives in Water Modulated by Thermoresponsive Dendritic Macromolecules

Jiaxing Zhang, Qinqin Ma, Huan Wang, Peinan Zhang, Xinyan Su,* Afang Zhang,* and Wen Li*

International Joint Laboratory of Biomimetic and Smart Polymers, School of Materials Science and Engineering, Shanghai University, Mailbox 152, Shangda Rd. 99, Shanghai 200444, China. Tel: +86-21-66138053. Fax: +86-21-66138039. Emails: wli@shu.edu.cn (W.L.), xysu@shu.edu.cn (X.S.), and azhang@shu.edu.cn (A.Z.)

Table of Contents

Figure S1. ^{1}H and ^{13}C NMR spectra of **Et-Dm** in $CDCl_3$....S3
Figure S2. ^{1}H and ^{13}C NMR spectra of **Me-Do** in $CDCl_3$....S4
Figure S3. ^{1}H and ^{13}C NMR spectra of **Et-Do** in $CDCl_3$....S5
Figure S4. ^{1}H spectra of **Poly(G1Et$_{20}$-*co*-Boc$_1$)** in $CDCl_3$....S6
Figure S5. ^{1}H spectra of **Poly(G1Me$_{20}$-*co*-Boc$_1$)** in $CDCl_3$....S6
Figure S6. ^{1}H spectra of **Poly(G1Et$_{20}$-*co*-H$_1$)** in $CDCl_3$....S7
Figure S7. ^{1}H spectra of **Poly(G1Me$_{20}$-*co*-H$_1$)** in $CDCl_3$....S7
Figure S8. ^{1}H and ^{13}C NMR spectra of **Poly(G1Et$_{20}$-*co*-Dm$_1$)** in $CDCl_3$....S8
Figure S9. ^{1}H and ^{13}C NMR spectra of **Poly(G1Me$_{20}$-*co*-Do$_1$)** in $CDCl_3$....S9
Figure S10. ^{1}H and ^{13}C NMR spectra of **Poly(G1Et$_{20}$-*co*-Do$_1$)** in $CDCl_3$....S10
Figure S11. HR-MS spectrum of compound **Et-Dm**....S11
Figure S12. HR-MS spectrum of compound **Me-Do**....S11
Figure S13. HR-MS spectrum of compound **Et-Do**....S11
Table S1. Conditions for and results from the copolymerization of **G1Me** or **G1Et** with **Dm** or **Do**....S12
Figure S14. Optical micrographs of the aqueous solutions from dendronized DASAs (a) and the corresponding dendronized copolymethacrylates (b) below and above their T_{cp}s....S12
Figure S15. UV/vis spectra of dendronized DASAs in aqueous solutions with a concentration of 0.25 mg·mL^{-1} (a), as well as these from **Et-Dm** (b), **Me-Do** (c), and **Et-Do** (d) in aqueous solutions with different concentrations....S13
Figure S16. Plots of time-dependent absorbance at λ_{max} for **Et-Dm** (a), **Me-Do** (b), and **Et-Do** (c) in aqueous solutions with different concentrations in dark....S14
Figure S17. UV/vis spectra of **Et-Dm** (a), **Me-Do** (b), and **Et-Do** (c) in aqueous solutions at different temperatures in dark....S14
Figure S18. UV/vis spectra of **Et-Dm** (concentration = 0.25 mg·mL^{-1}) photoisomerization (a), light-sheltered heating at 25 °C (b), 45 °C (c), and 65 °C (d)....S15
Figure S19. UV/vis spectra of **Me-Do** (concentration = 0.25 mg·mL^{-1}) photoisomerization (a), light-sheltered heating at 25 °C (b), 45 °C (c), and 65 °C (d)....S15
Figure S20. UV/vis spectra of **Et-Do** (concentration = 0.25 mg·mL^{-1}) photoisomerization (a), light-sheltered heating at 25 °C (b), 45 °C (c), and 65 °C (d)....S16
Figure S21. Plots of R_h *vs* temperature for **Et-Dm**, **Me-Do**, and **Et-Do** at different temperatures (a), and plots of R_h *vs* temperature for **Me-Do** and **Et-Do** in aqueous solutions after repeated photo-irradiation and heating (b). Concentration = 0.25 mg·mL^{-1}....S16
Figure S22. Plots of absorbance at λ_{max} for **Et-Dm** (a), **Me-Do** (b), and **Et-Do** (c) with different concentrations against time after photo-irradiation and followed by annealing at 65 °C....S17
Figure S23. UV/vis spectra of **Poly(G1Et$_{20}$-*co*-Dm$_1$)**, **Poly(G1Me$_{20}$-*co*-Do$_1$)**, and **Poly(G1Et$_{20}$-*co*-Do$_1$)** (c) in aqueous solutions with different concentrations....S17
Figure S24. UV/vis spectra of **Poly(G1Et$_{20}$-*co*-Dm$_1$)** (a), **Poly(G1Me$_{20}$-*co*-Do$_1$)** (b), and **Poly(G1Et$_{20}$-*co*-Do$_1$)** (c) in aqueous solutions at different temperatures in dark....S18
Figure S25. UV/vis spectra of **Poly(G1Et$_{20}$-*co*-Dm$_1$)** in aqueous solutions (concentration = 0.25 mg·mL^{-1}) through photo-irradiation (a), and followed by thermal annealing at 25 °C (b), 45 °C (c), and 65 °C (d) in dark....S18
Figure S26. UV/vis spectra of **Poly(G1Me$_{20}$-*co*-Do$_1$)** in aqueous solutions (concentration = 0.25 mg·mL^{-1}) through photo-irradiation (a), and followed by thermal annealing at 25 °C (b), 45 °C (c), and 65 °C (d) in dark....S19
Figure S27. UV/vis spectra of **Poly(G1Et$_{20}$-*co*-Do$_1$)** in aqueous solutions (concentration = 0.25 mg·mL^{-1}) through photo-irradiation (a), and followed by thermal annealing at 25 °C (b), 45 °C (c), and 65 °C (d) in dark....S19

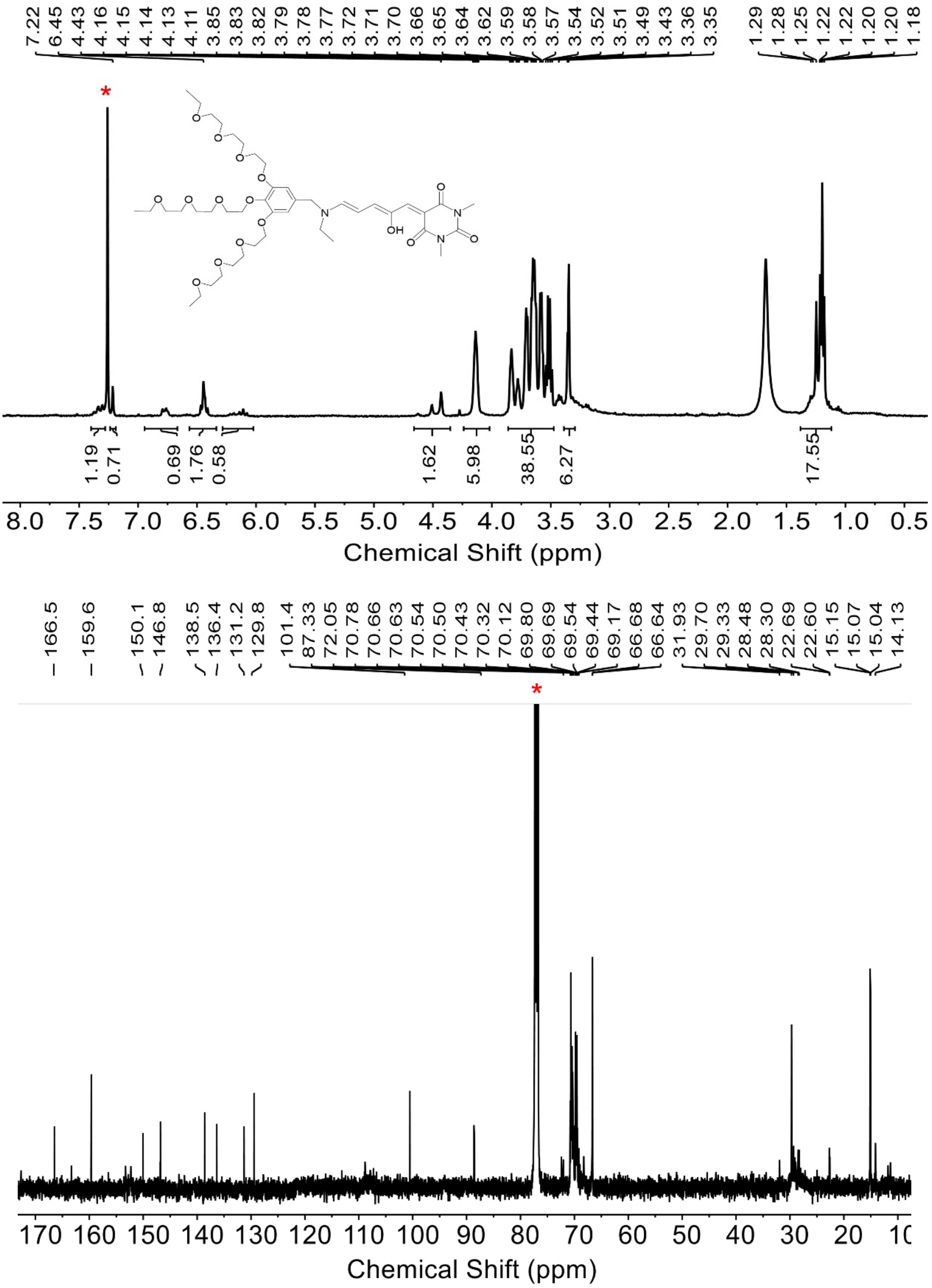

Figure S1. ^{1}H and ^{13}C NMR spectra of **Et-Dm** in $CDCl_3$. Solvent peaks and water peaks marked as *.

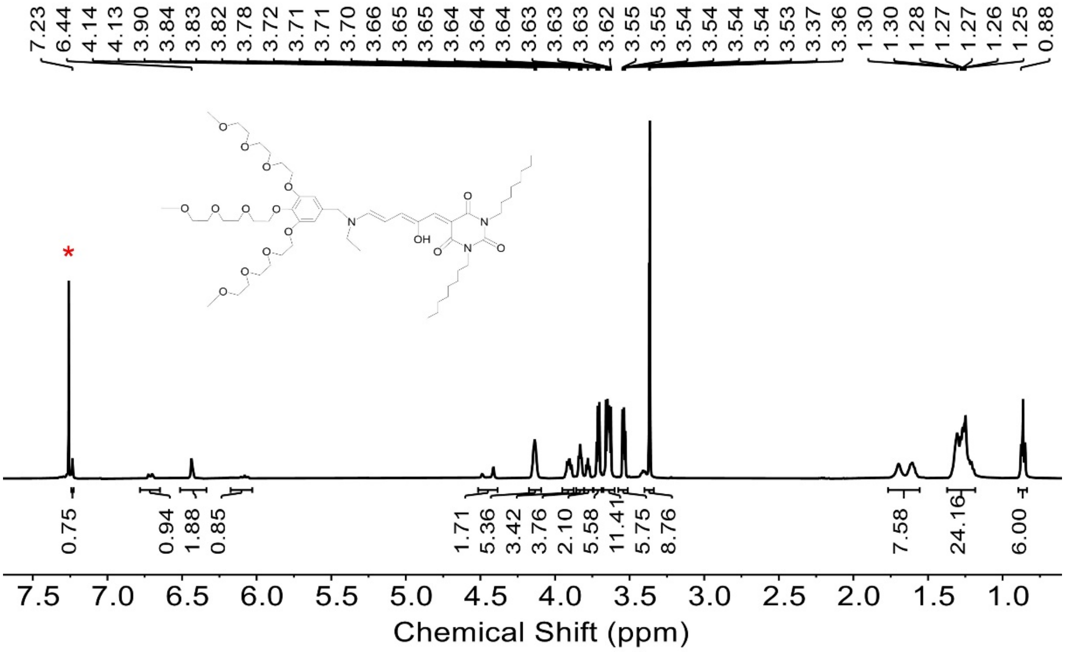

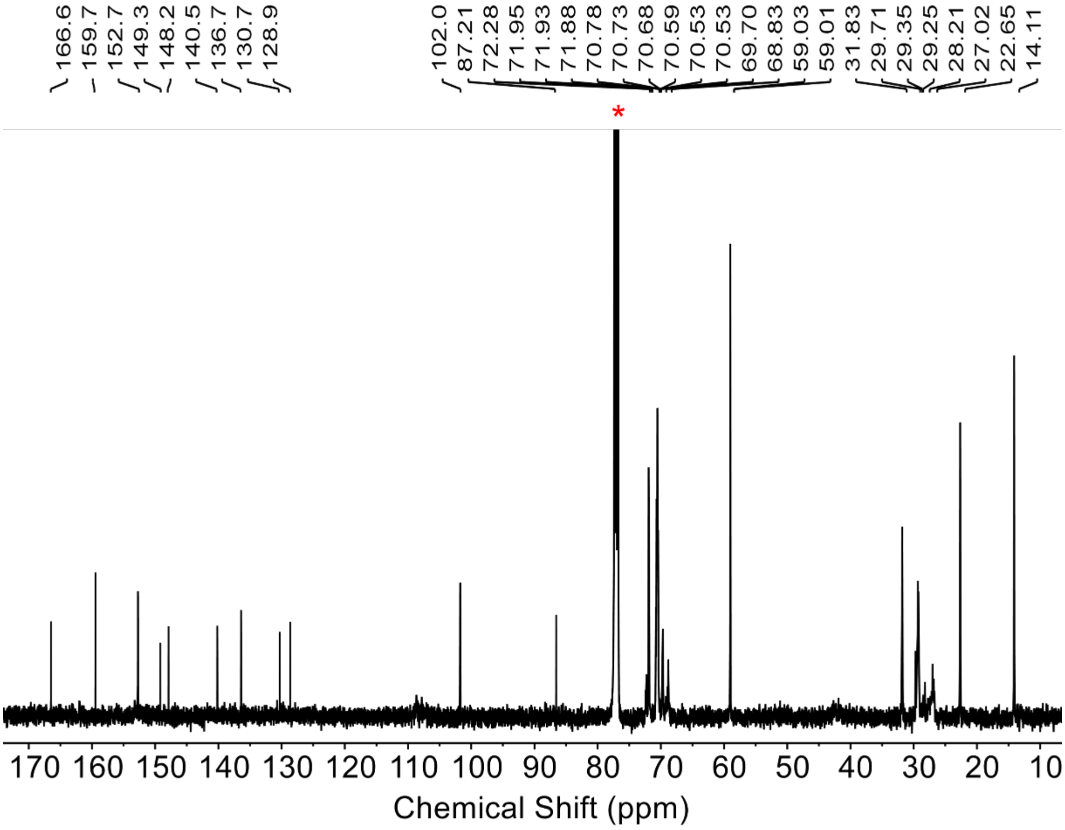

Figure S2. ^{1}H and ^{13}C NMR spectra of **Me-Do** in $CDCl_3$. Solvent peaks and water peaks marked as *.

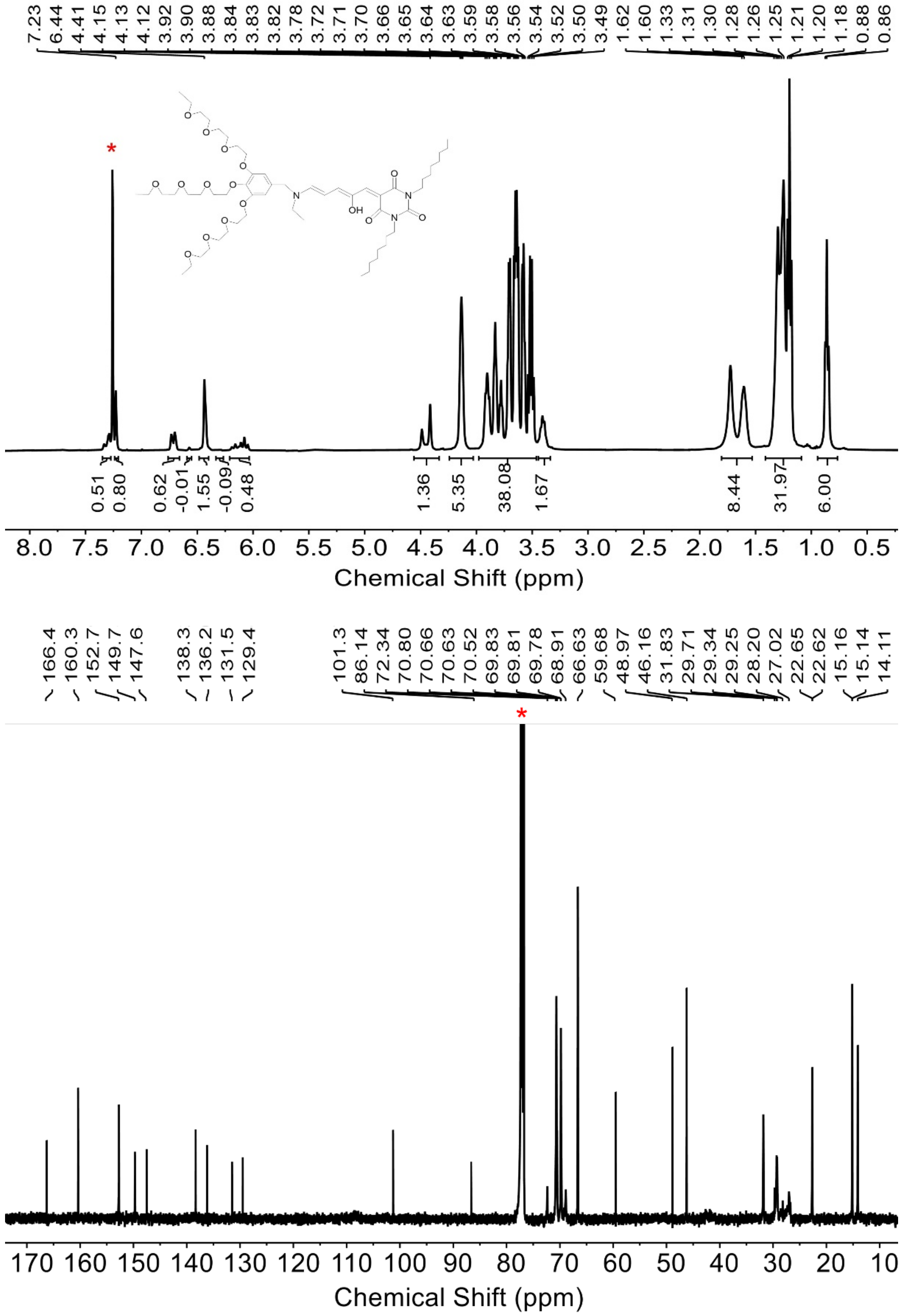

Figure S3. ^{1}H and ^{13}C NMR spectra of **Et-Do** in $CDCl_3$. Solvent peaks and water peaks marked as *.

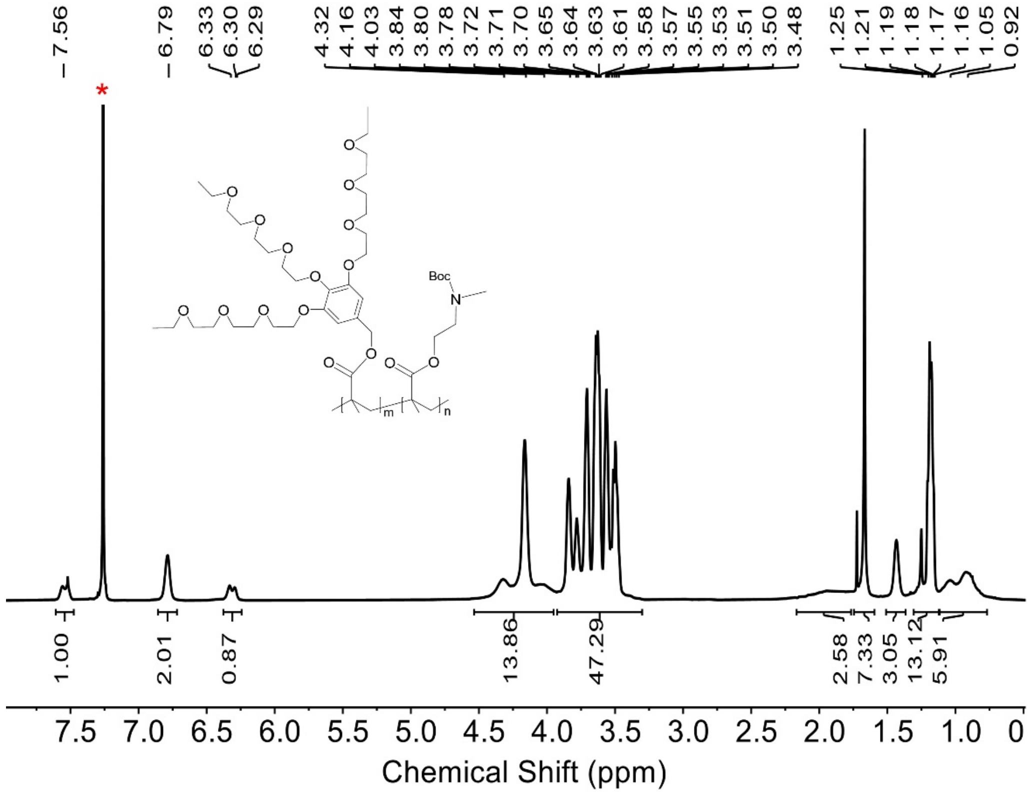

Figure S4. ^{1}H spectra of **Poly(G1Et$_{20}$-*co*-Boc$_1$)** in $CDCl_3$. Solvent peaks and water peaks are marked as *.

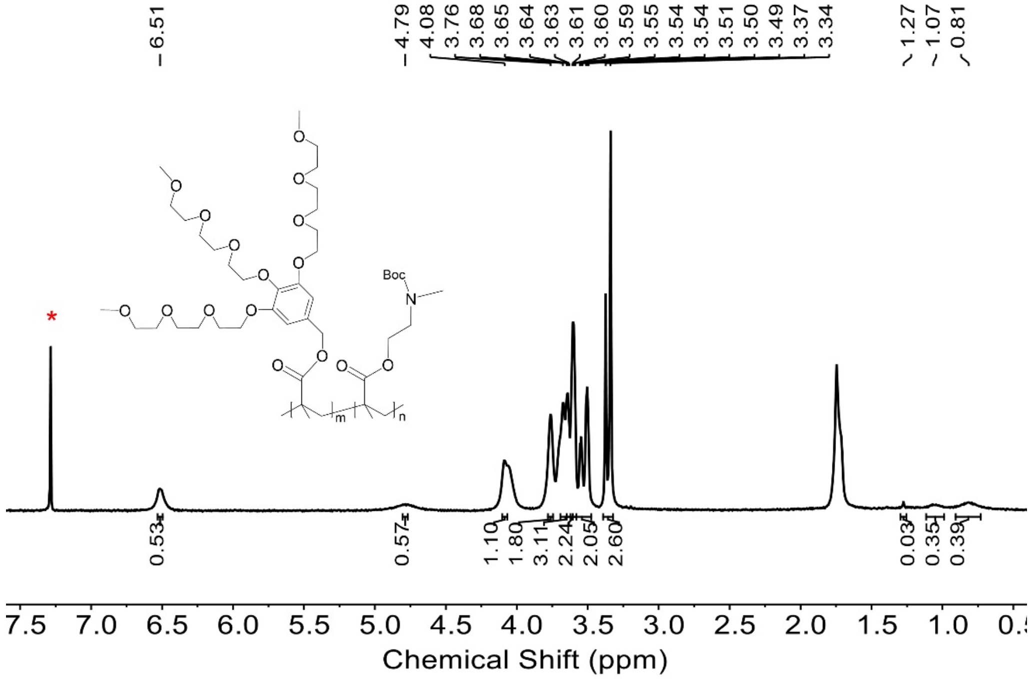

Figure S5. ^{1}H spectra of **Poly(G1Me$_{20}$-*co*-Boc$_1$)** in $CDCl_3$. Solvent peaks and water peaks are marked as *.

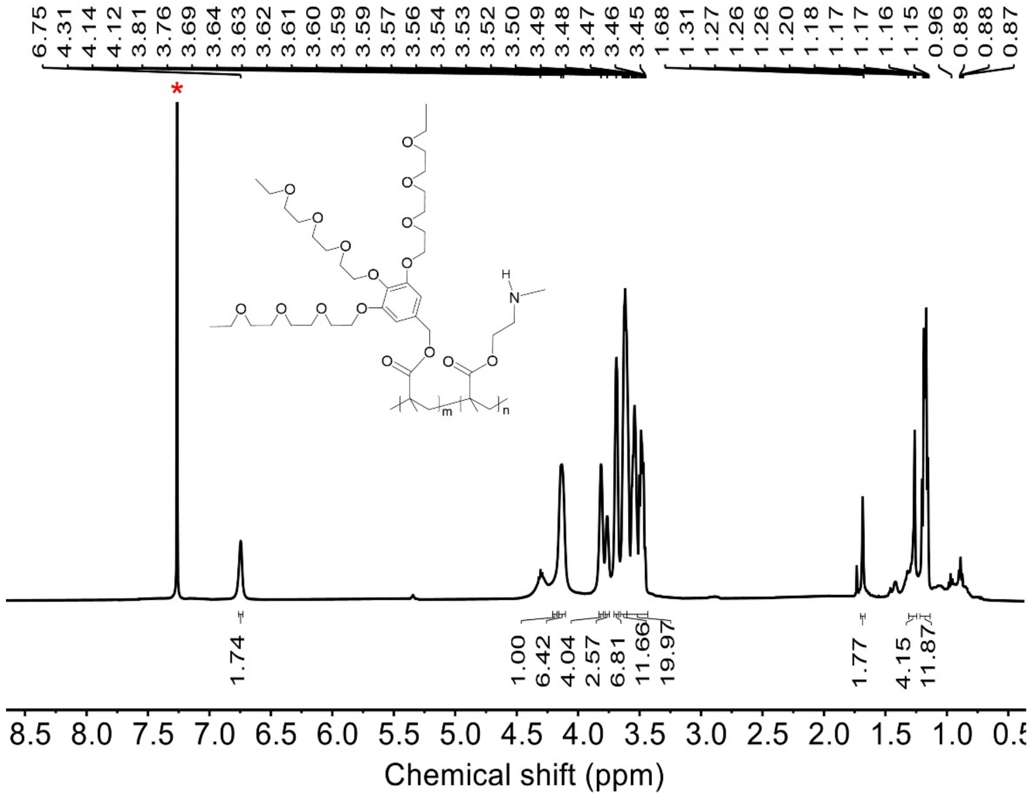

Figure S6. ^{1}H spectra of **Poly(G1Et$_{20}$-*co*-H$_1$)** in $CDCl_3$. Solvent peaks and water peaks are marked as *.

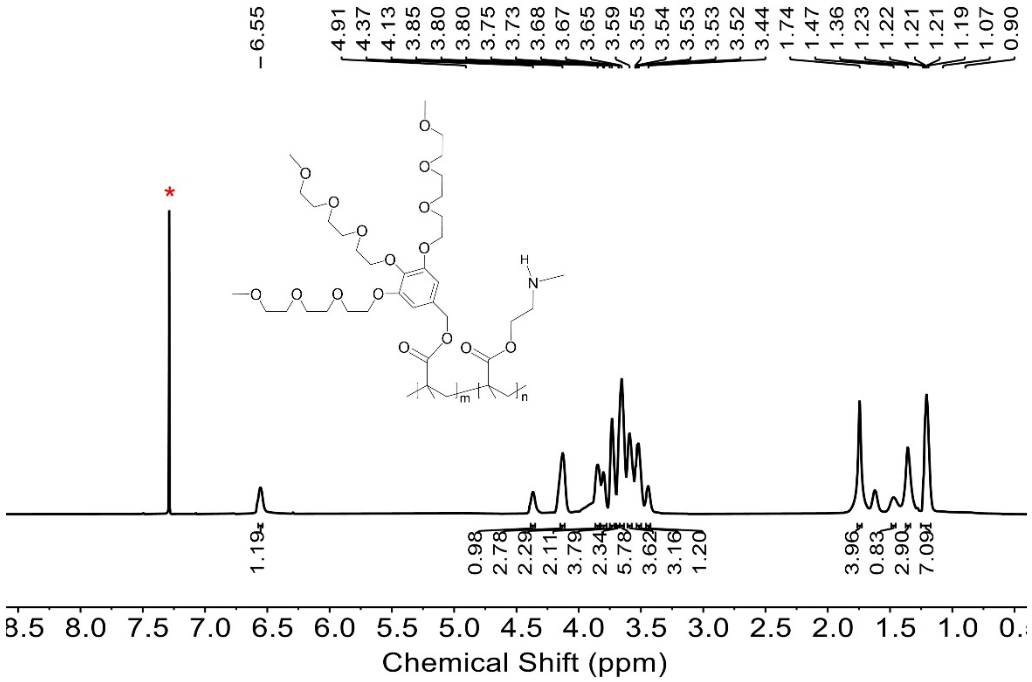

Figure S7. ^{1}H spectra of **Poly(G1Me$_{20}$-*co*-H$_1$)** in $CDCl_3$. Solvent peaks and water peaks are marked as *.

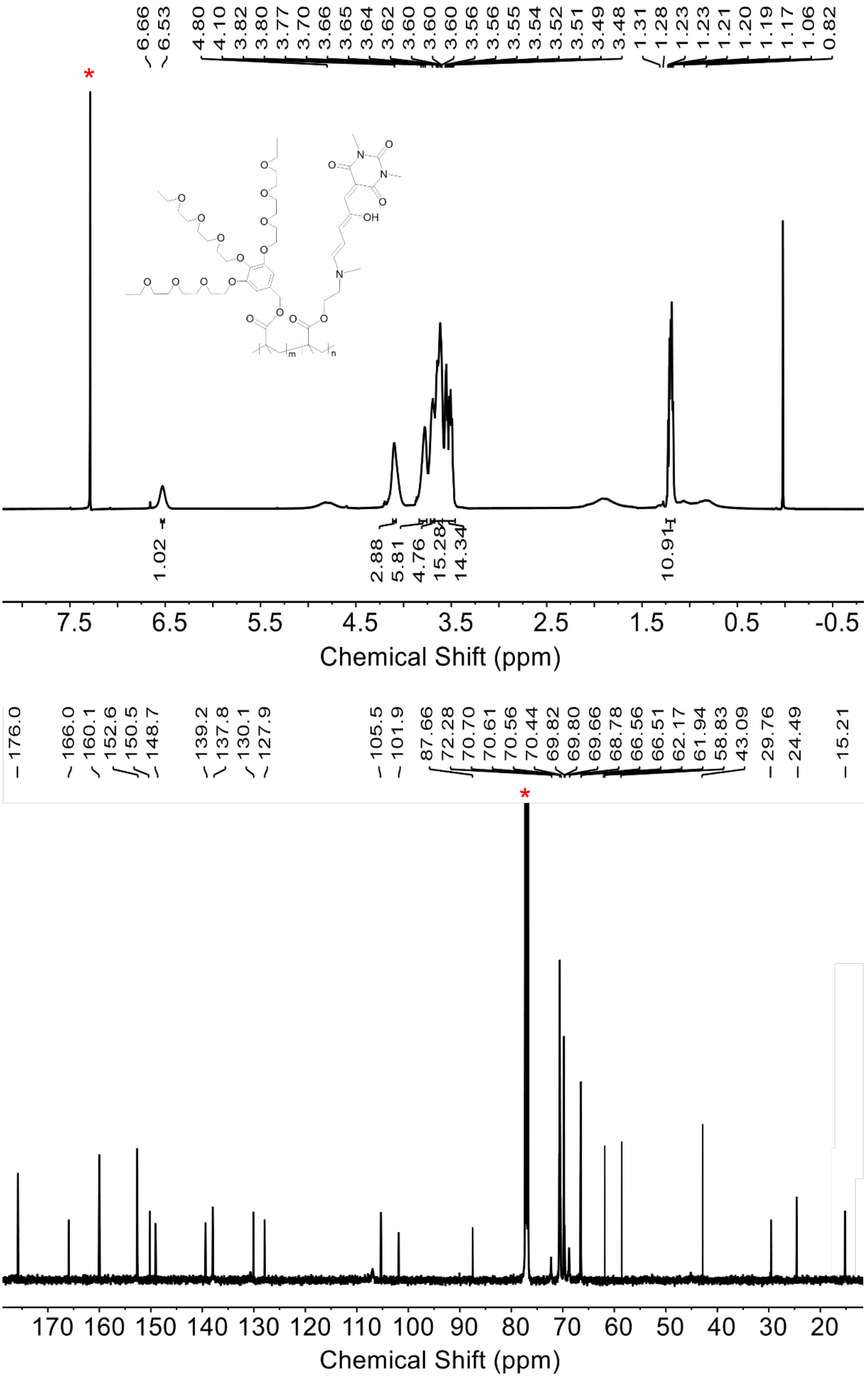

Figure S8. ^{1}H and ^{13}C NMR spectra of **Poly(G1Et$_{20}$-*co*-Dm$_{1}$)** in $CDCl_3$. Solvent peaks and water peaks are marked as *.

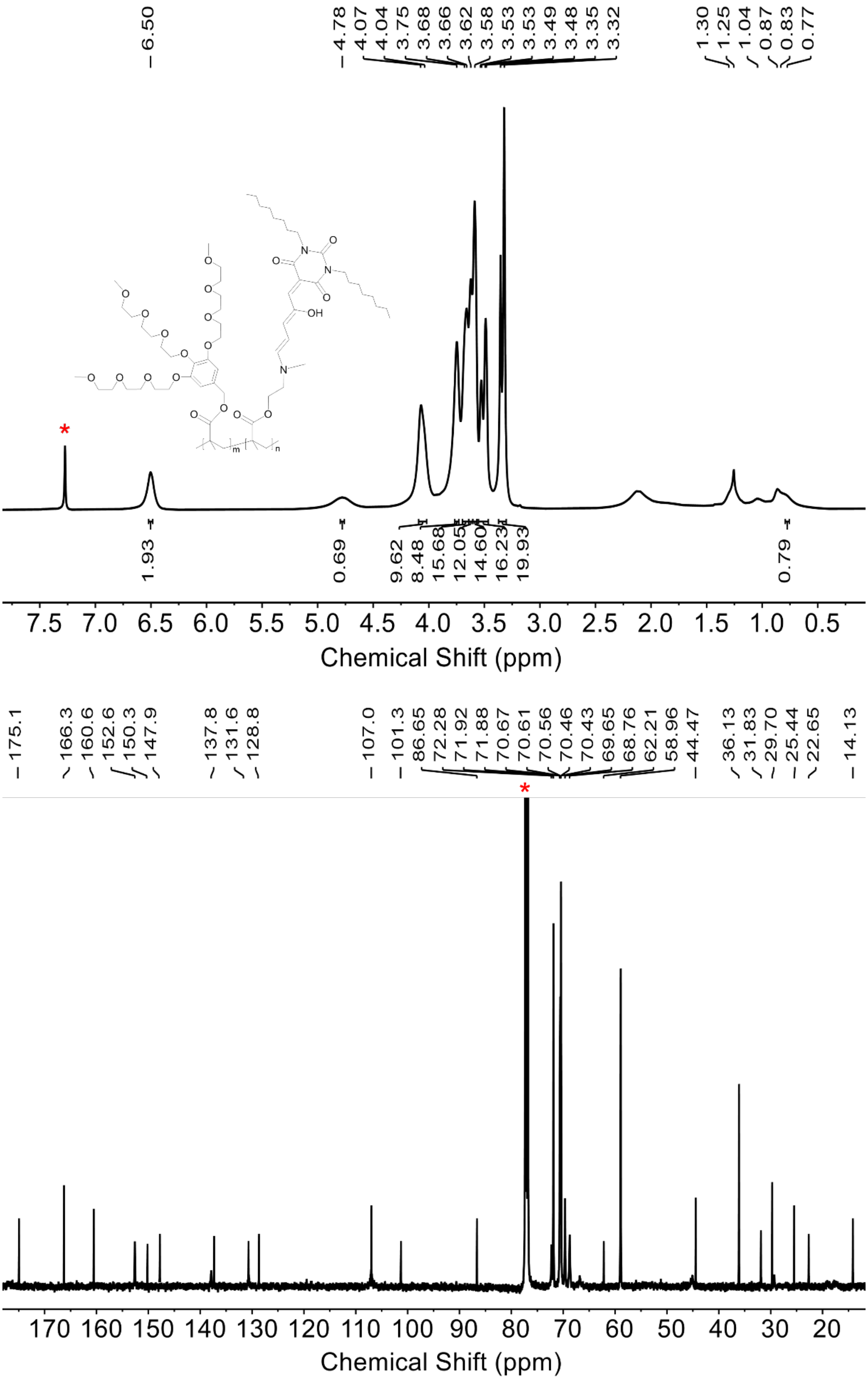

Figure S9. ^{1}H and ^{13}C NMR spectra of **Poly(G1Me$_{20}$-*co*-Do$_{1}$)** in $CDCl_3$. Solvent peaks and water peaks are marked as *.

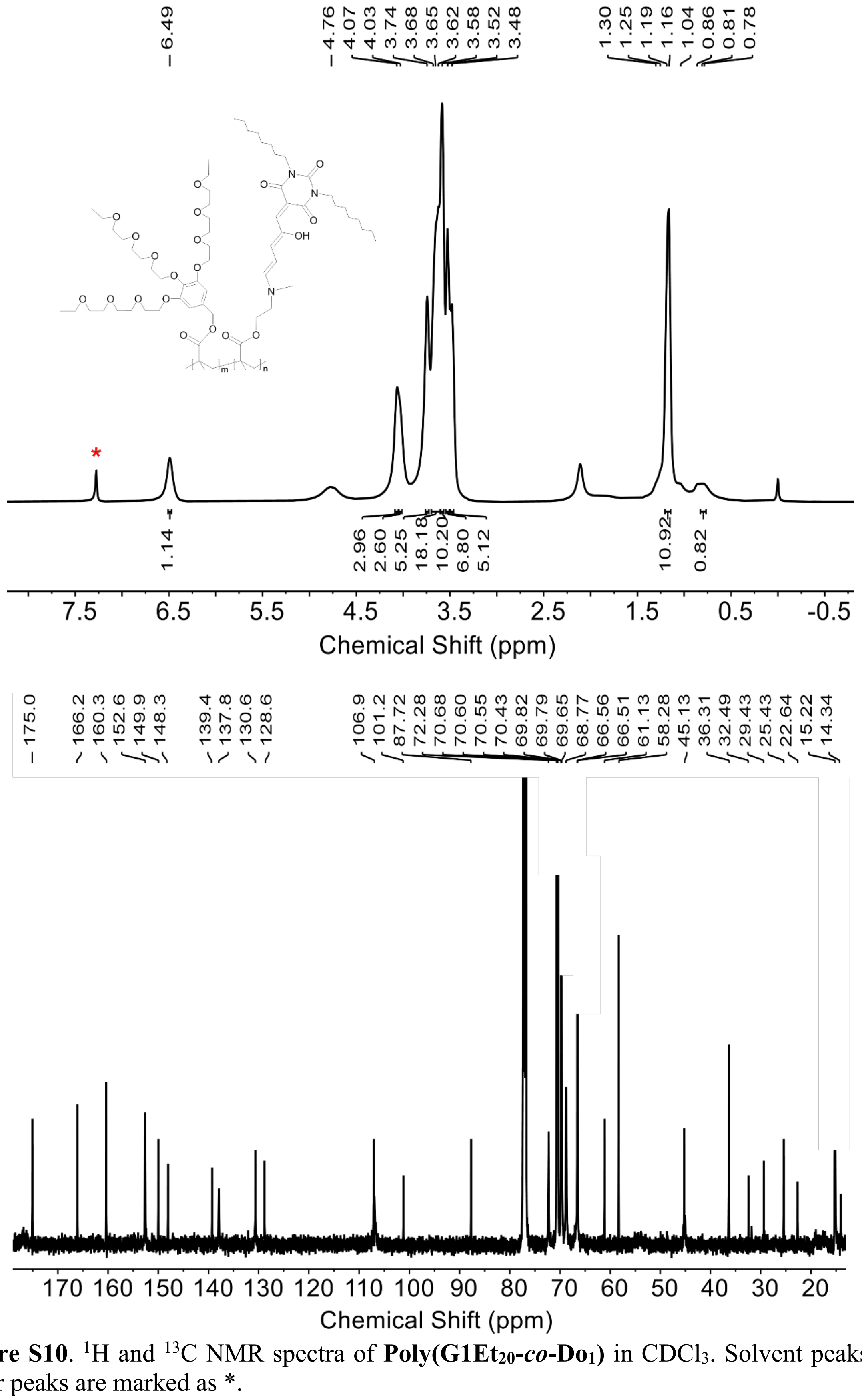

Figure S10. ^{1}H and ^{13}C NMR spectra of **Poly(G1Et$_{20}$-*co*-Do$_{1}$)** in $CDCl_3$. Solvent peaks and water peaks are marked as *.

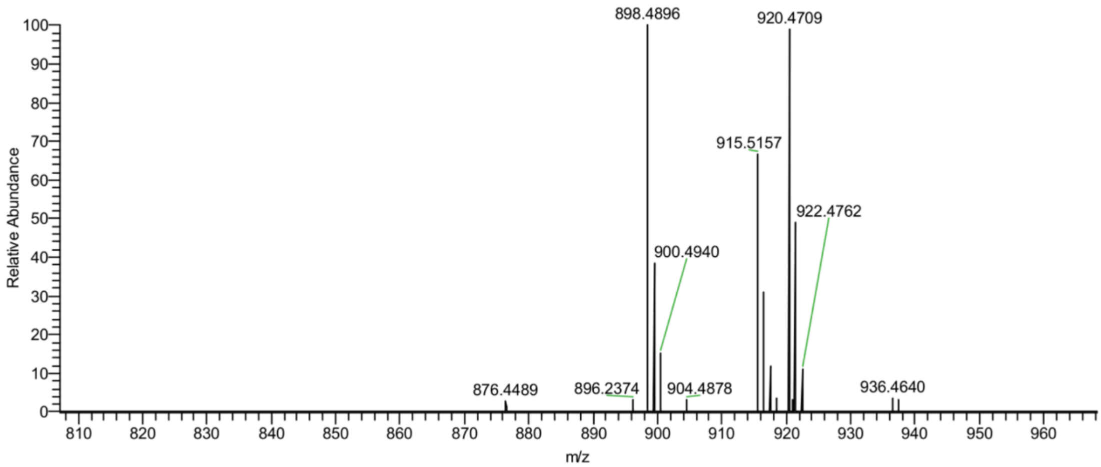

Figure S11. HR-MS spectrum of compound **Et-Dm**.

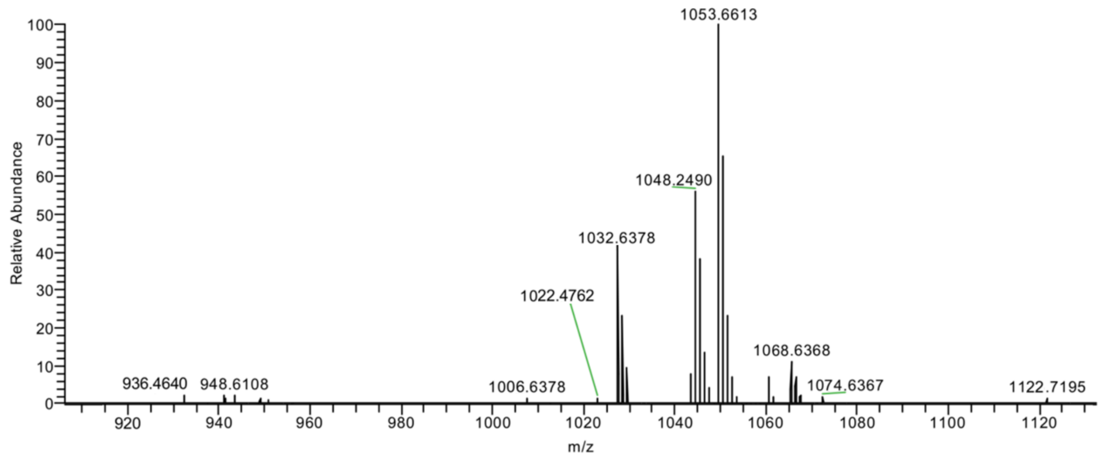

Figure S12. HR-MS spectrum of compound **Me-Do**.

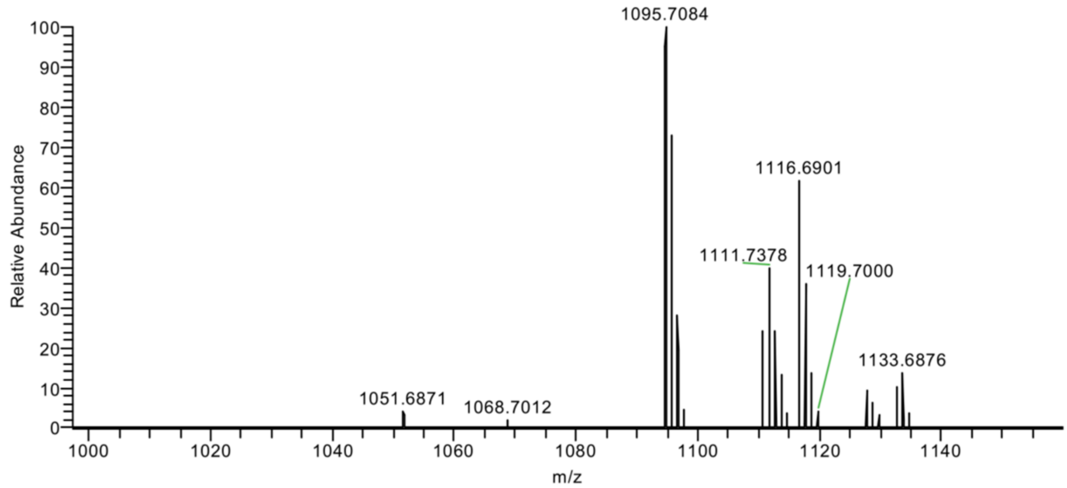

Figure S13. HR-MS spectrum of compound **Et-Do**.

Table S1. Conditions for and results from the copolymerization of **G1Me** or **G1Et** with **Dm** or **Do**.

Sample names	Feed ratio [a]	Actual ratio [b]	GPC result [c]	
			M_n (×10^{-5})	*Đ*
**Poly(G1Et$_{20}$-*co*-Dm$_1$)**	20:1	20:1	1.1	2.5
**Poly(G1Me$_{20}$-*co*-Do$_1$)**	20:1	22:1	1.3	2.7
**Poly(G1Et$_{20}$-*co*-Do$_1$)**	20:1	21:1	1.0	3.1

[a] Feed ratio of monomer **G1Me** or **G1Et** to monomer **Dm** or **Do**. [b] Obtained from the proton integral ratios of ^{1}H NMR spectra. [c] Determined by GPC with DMF as eluent containing 1.00 mg·mL^{-1} of LiBr. M_n and *Đ* represent number-average molecular weight and polydispersity of the polymers, respectively.

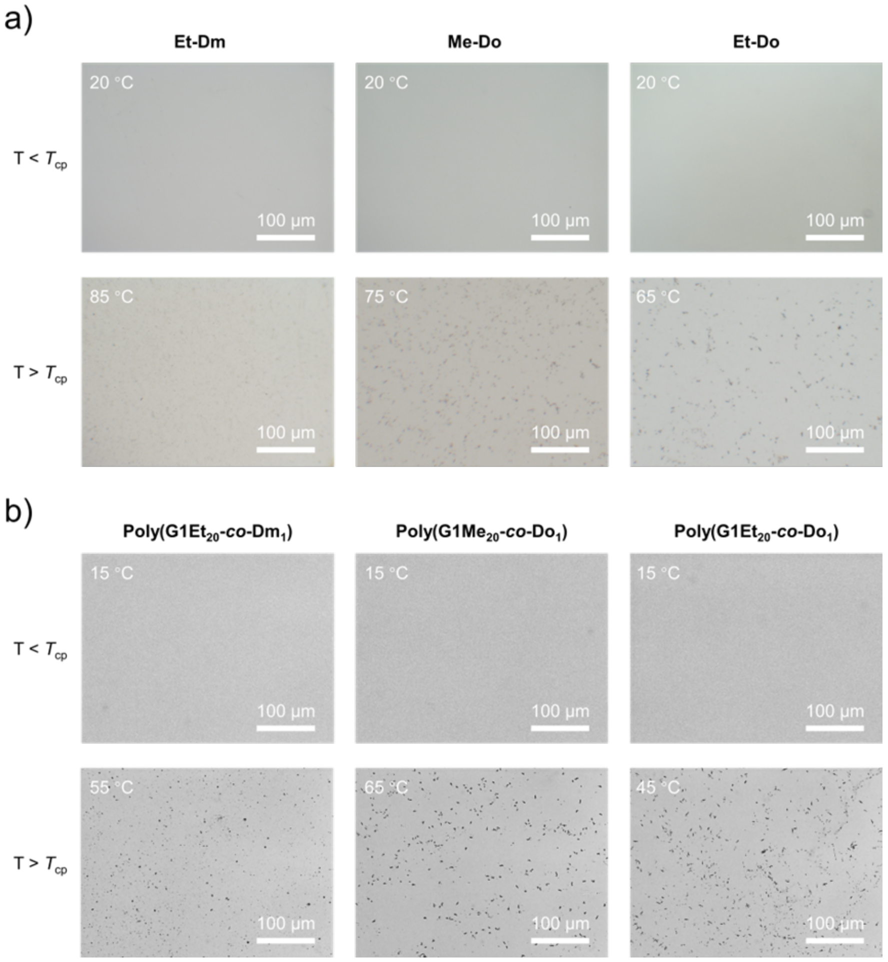

Figure S14. Optical micrographs of the aqueous solutions from dendronized DASAs (a) and the corresponding dendronized copolymethacrylates (b) below and above their T_{cp}s. Concentration = 0.50 mg·mL^{-1}.

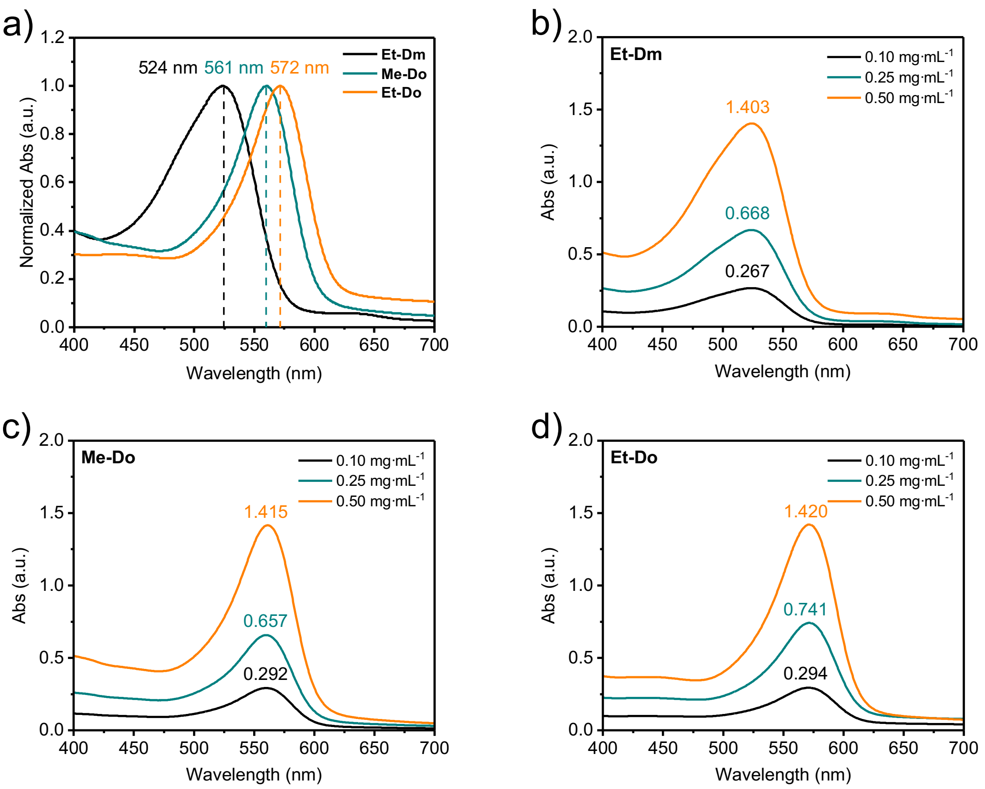

Figure S15. UV/vis spectra of the dendronized DASAs in aqueous solutions with a concentration of 0.25 mg·mL^{-1} (a), as well as these from **Et-Dm** (b), **Me-Do** (c), and **Et-Do** (d) in aqueous solutions with different concentrations. Temperature was set to 25.0 °C for all measurements.

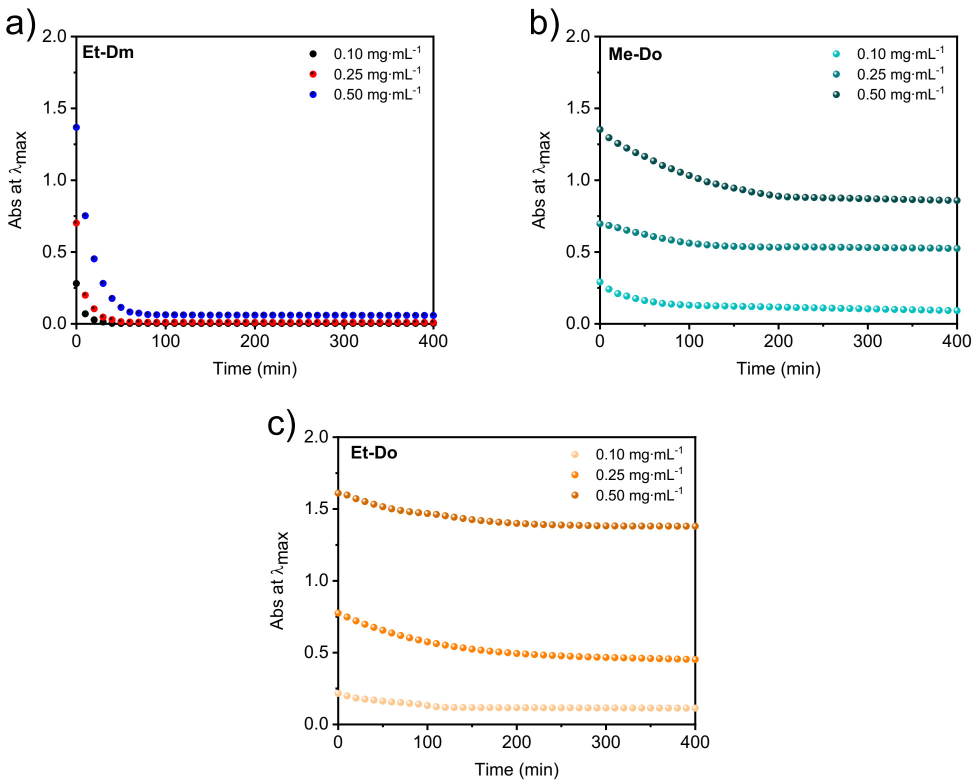

Figure S16. Plots of time-dependent absorbance at λ_{max} for **Et-Dm** (a), **Me-Do** (b), and **Et-Do** (c) in aqueous solutions with different concentrations in dark.

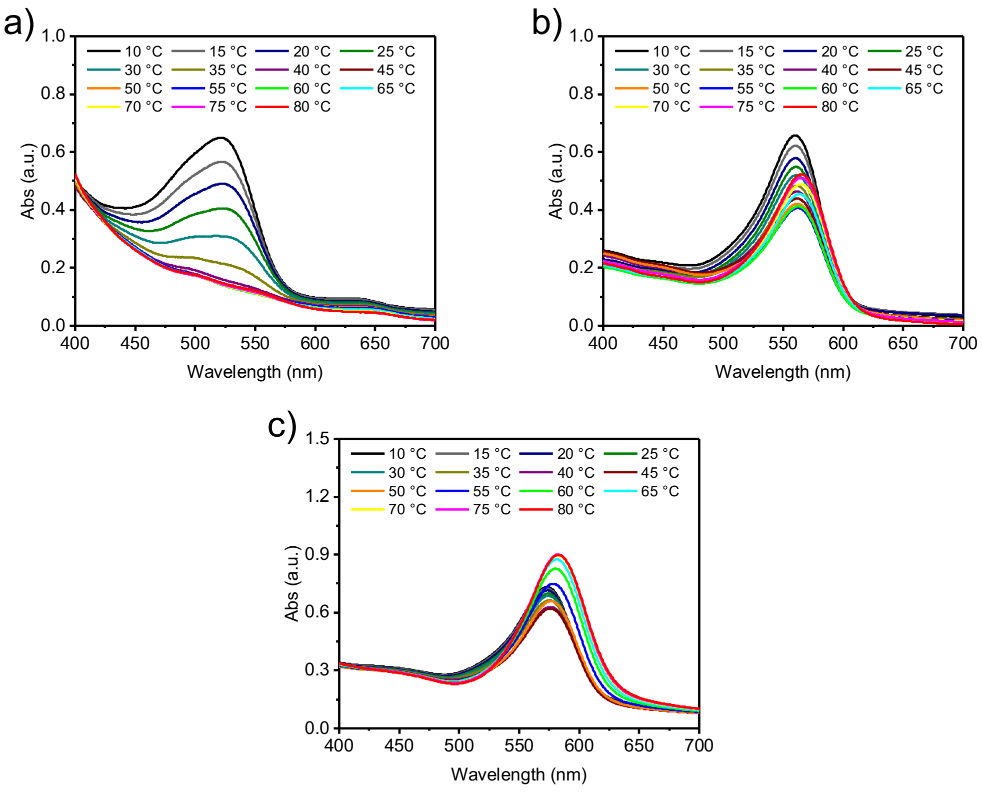

Figure S17. UV/vis spectra of **Et-Dm** (a), **Me-Do** (b), and **Et-Do** (c) in aqueous solutions at different temperatures in dark. Concentration = 0.25 mg·mL^{-1}.

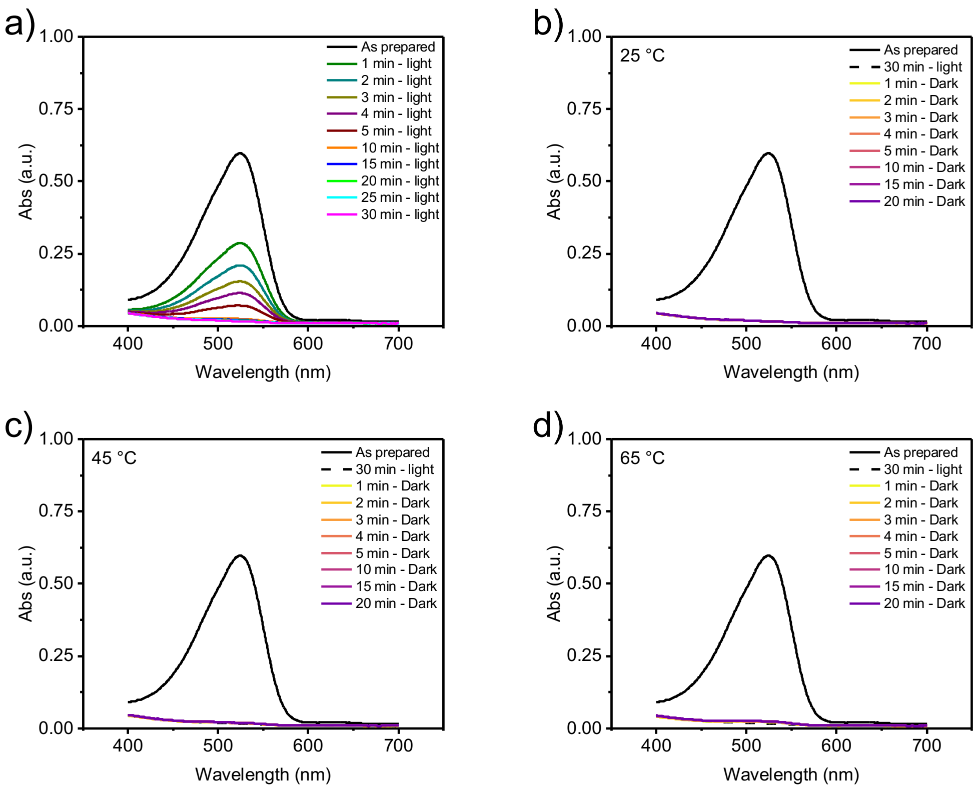

Figure S18. UV/vis spectra of **Et-Dm** (concentration = 0.25 mg·mL^{-1}) photoisomerization (a), light-sheltered heating at 25 °C (b), 45 °C (c), and 65 °C (d).

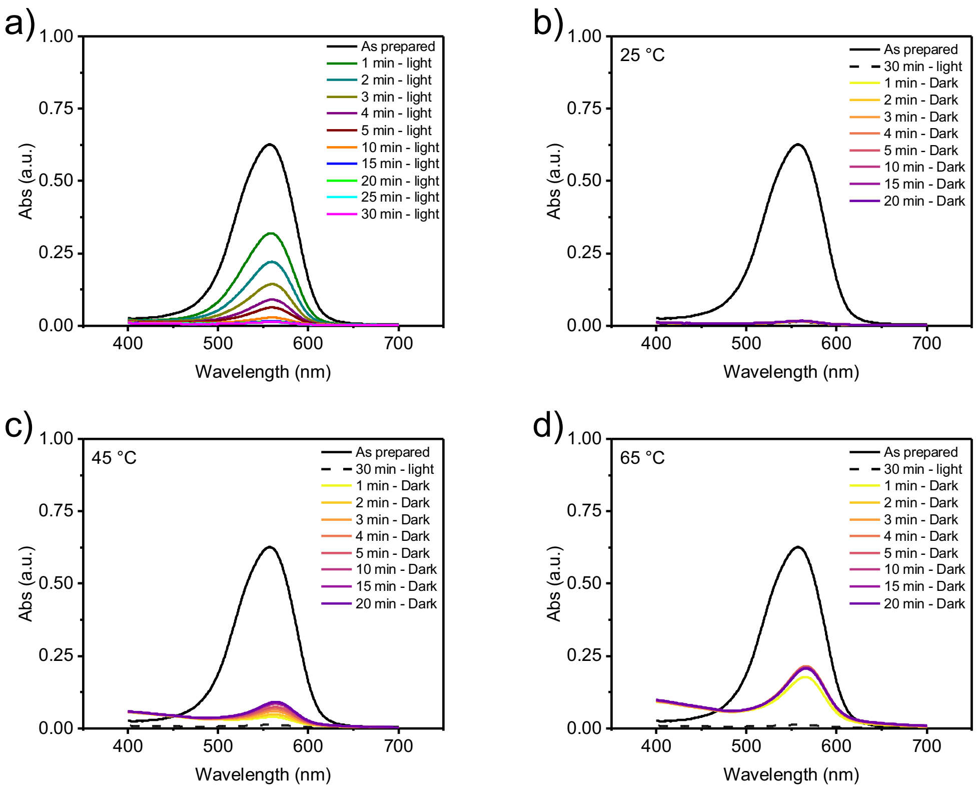

Figure S19. UV/vis spectra of **Me-Do** (concentration = 0.25 mg·mL^{-1}) photoisomerization (a), light-sheltered heating at 25 °C (b), 45 °C (c), and 65 °C (d).

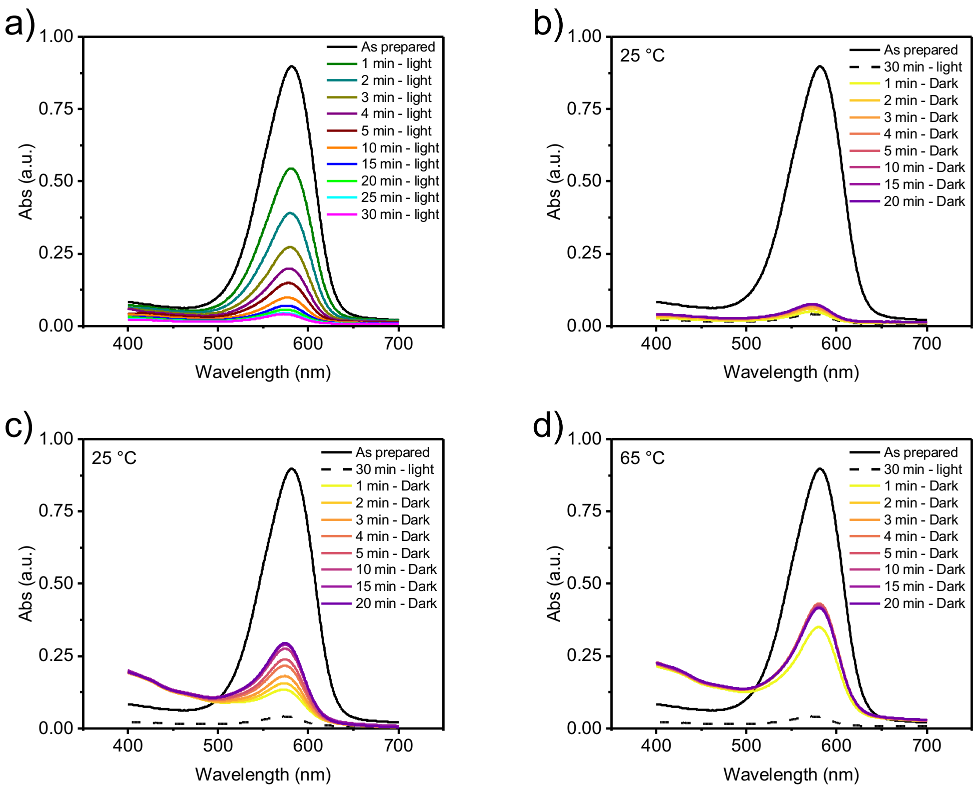

Figure S20. UV/vis spectra of **Et-Do** (concentration = 0.25 mg·mL^{-1}) photoisomerization (a), light-sheltered heating at 25 °C (b), 45 °C (c), and 65 °C (d).

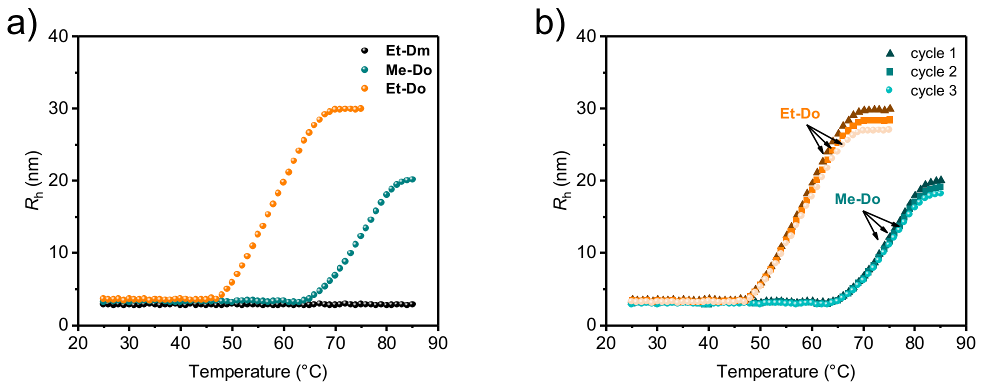

Figure S21. Plots of R_h *vs* temperature for **Et-Dm**, **Me-Do**, and **Et-Do** at different temperatures (a), and plots of R_h *vs* temperature for **Me-Do** and **Et-Do** in aqueous solutions after repeated photo-irradiation and heating (b). Concentration = 0.25 mg·mL^{-1}.

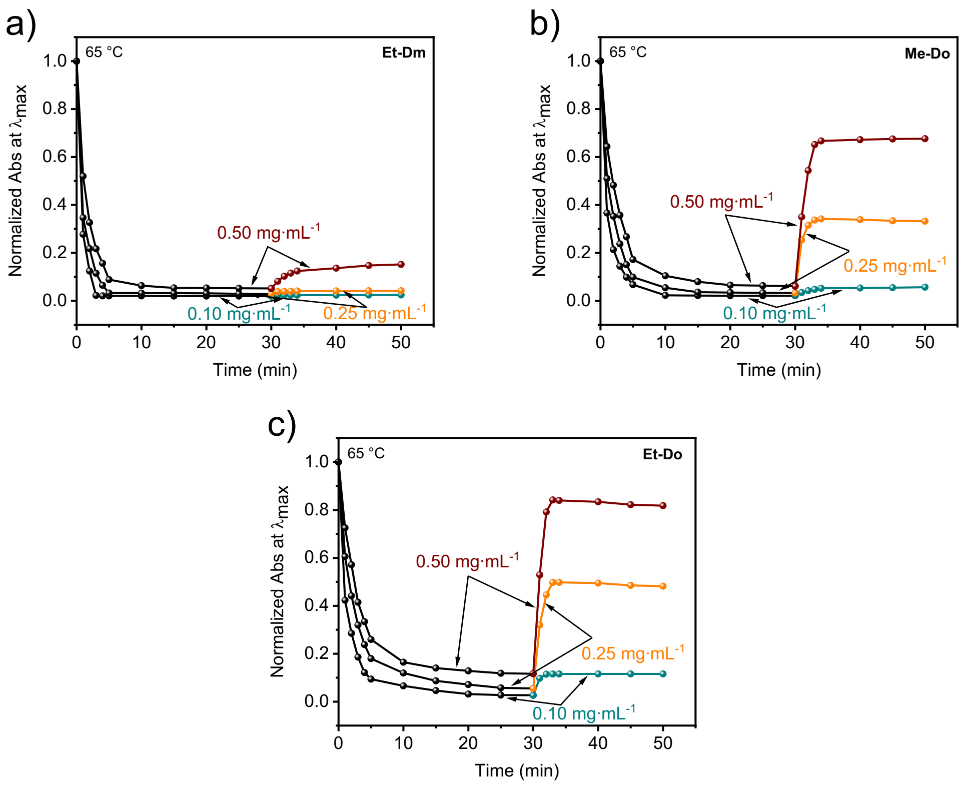

Figure S22. Plots of absorbance at λ_{max} for **Et-Dm** (a), **Me-Do** (b), and **Et-Do** (c) with different concentrations against time after photo-irradiation and followed by annealing at 65 °C. The absorbance was normalized.

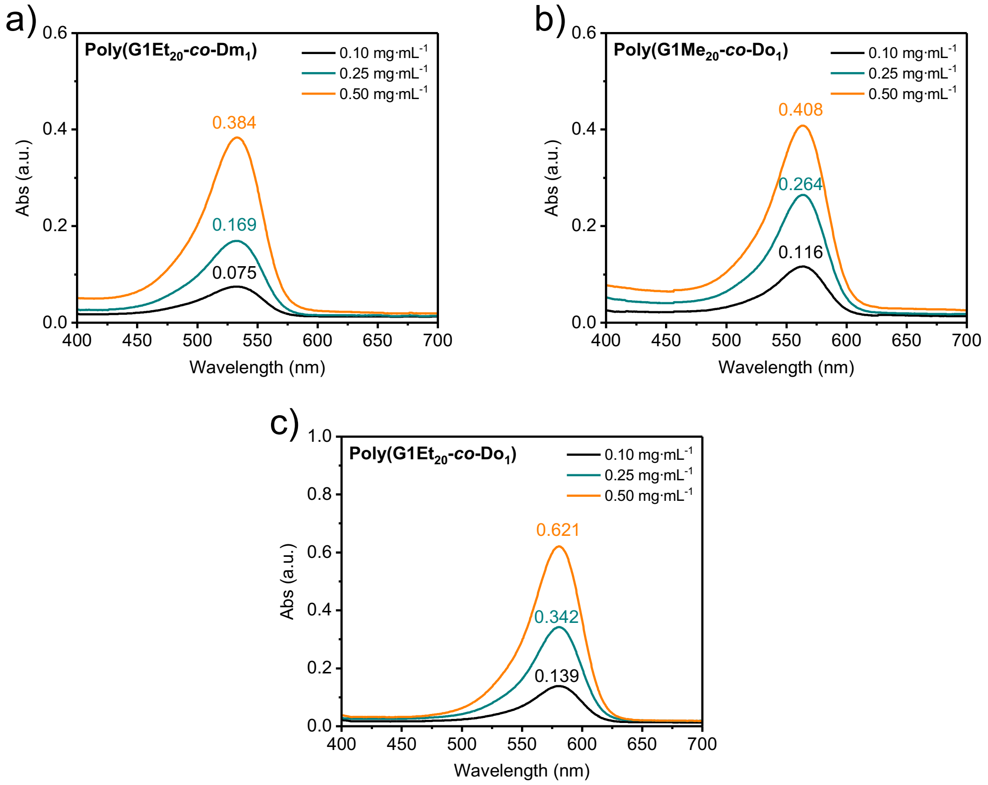

Figure S23. UV/vis spectra of **Poly(G1Et$_{20}$-*co*-Dm$_1$)** (a), **Poly(G1Me$_{20}$-*co*-Do$_1$)** (b), and **Poly(G1Et$_{20}$-*co*-Do$_1$)** (c) in aqueous solutions with different concentrations. Temperature was set to 25.0 °C for all measurements.

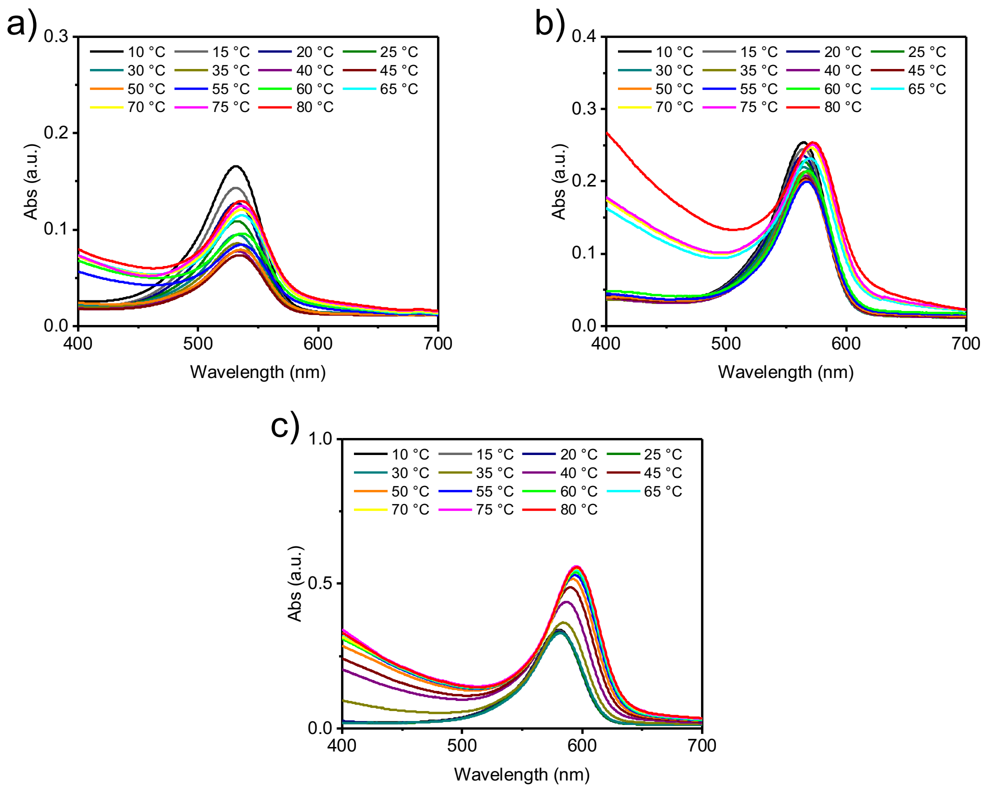

Figure S24. UV/vis spectra of **Poly(G1Et$_{20}$-*co*-Dm$_1$)** (a), **Poly(G1Me$_{20}$-*co*-Do$_1$)** (b), and **Poly(G1Et$_{20}$-*co*-Do$_1$)** (c) in aqueous solutions at different temperatures in dark. Concentration = 0.25 mg·mL^{-1}.

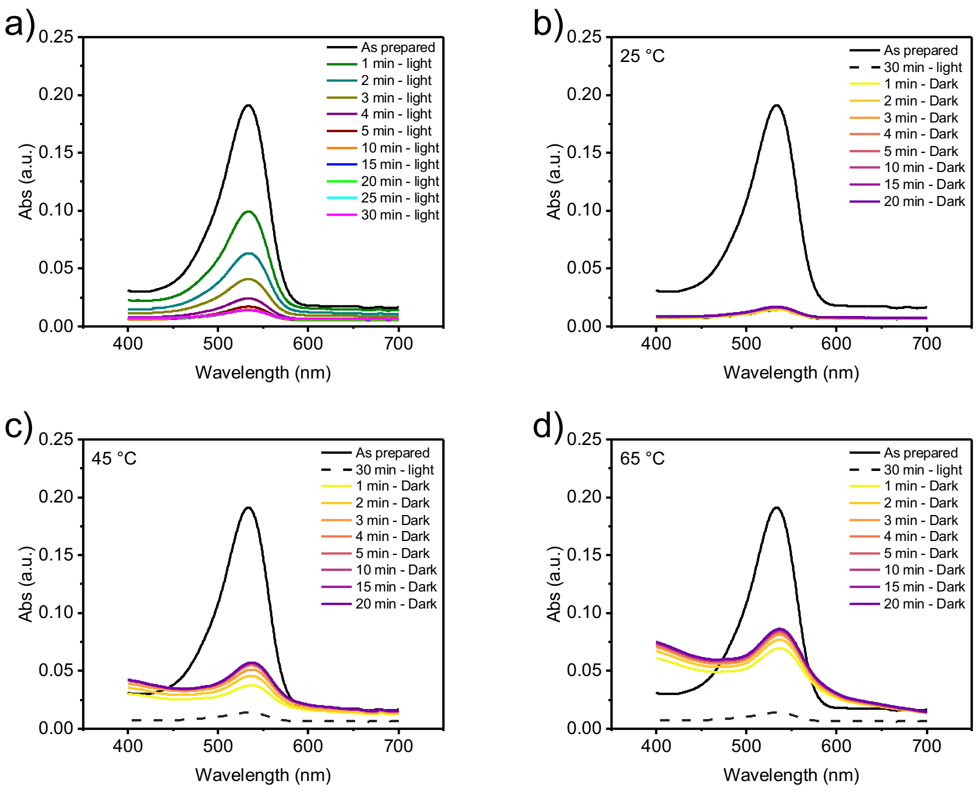

Figure S25. UV/vis spectra of **Poly(G1Et$_{20}$-*co*-Dm$_1$)** in aqueous solutions (concentration = 0.25 mg·mL^{-1}) through photo-irradiation (a), and followed by thermal annealing at 25 °C (b), 45 °C (c), and 65 °C (d) in dark.

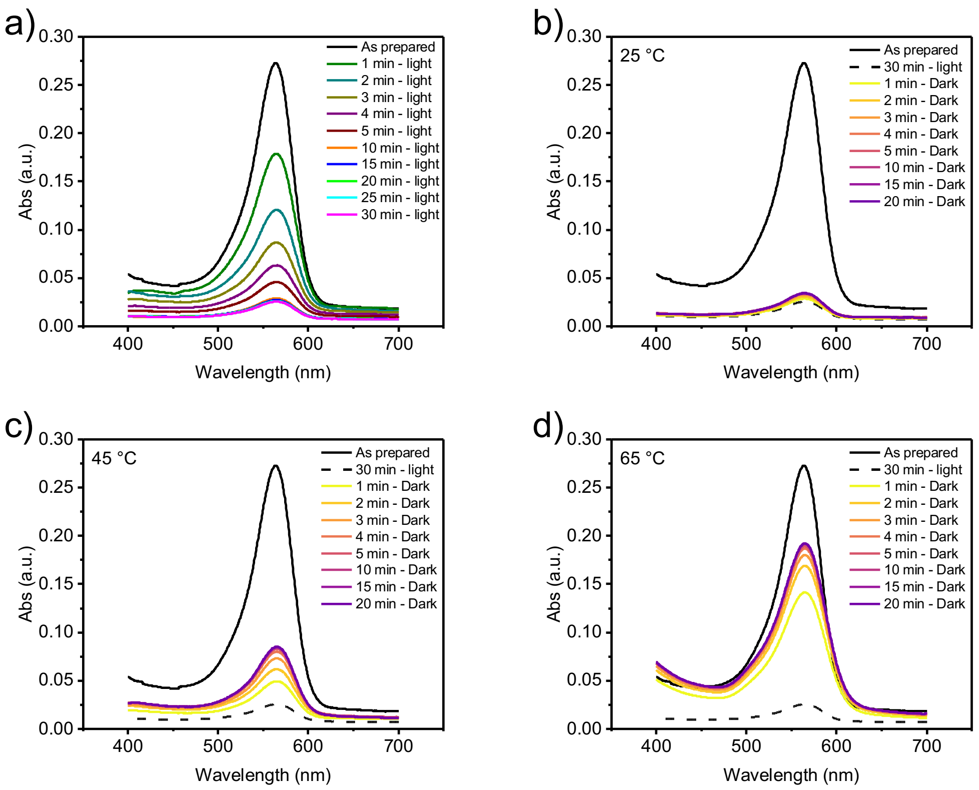

Figure S26. UV/vis spectra of **Poly(G1Me$_{20}$-*co*-Do$_1$)** in aqueous solutions (concentration = 0.25 mg·mL^{-1}) through photo-irradiation (a), and followed by thermal annealing at 25 °C (b), 45 °C (c), and 65 °C (d) in dark.

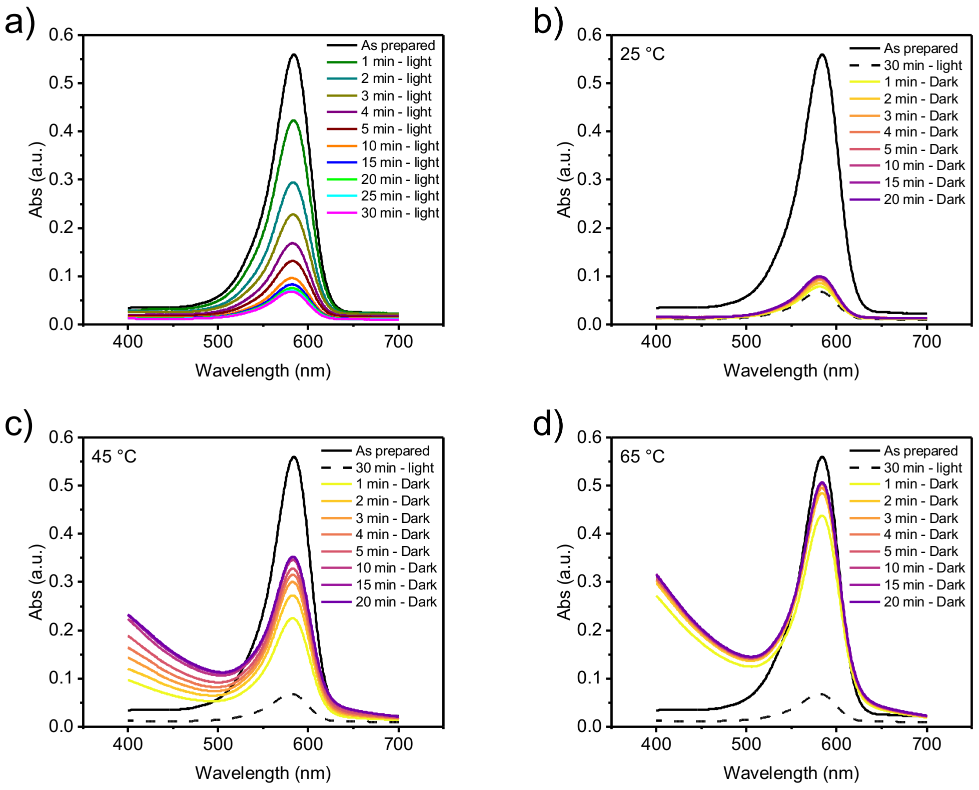

Figure S27. UV/vis spectra of **Poly(G1Et$_{20}$-*co*-Do$_1$)** in aqueous solutions (concentration = 0.25 mg·mL^{-1}) through photo-irradiation (a), and followed by thermal annealing at 25 °C (b), 45 °C (c), and 65 °C (d) in dark.
